# Effects of elexacaftor/tezacaftor/ivacaftor on the nasal microbial metagenome in cystic fibrosis

**DOI:** 10.1128/spectrum.00601-26

**Published:** 2026-07-13

**Authors:** Tom L. Hertramph, Marie Dorda, Sophia T. Pallenberg, Annette Sauer-Heilborn, Felix C. Ringshausen, Matthias Steglich, Gesine Hansen, Burkhard Tümmler, Lutz Wiehlmann, Ilona Rosenboom, Anna-Maria Dittrich

**Affiliations:** 1 Department for Pediatric Pneumology, Allergology and Neonatology, Hannover Medical School (MHH), Hannover, Germany; 2 DigiStrucMed Program, Dean’s Office for Academic Career Development, Hannover Medical School (MHH) 9177, Hannover, Germany; 3 Research Core Unit Genomics, Hannover Medical School (MHH) 9177, Hannover, Germany; 4 Department of Respiratory Medicine and Infectious Diseases, Hannover Medical School (MHH) 9177, Hannover, Germany; 5 German Center for Lung Research, Biomedical Research in Endstage and Obstructive Lung Disease Hannover (BREATH), Hannover Medical School (MHH) 9177, Hannover, Germany; 6 Cluster of Excellence RESIST (EXC 2155), German Research Foundation (DFG), Hannover Medical School (MHH) 9177, Hannover, Germany; Nova Southeastern University, Fort Lauderdale, Florida, USA

**Keywords:** CFTR modulation, ELX/TEZ/IVA, nasal metagenome, cystic fibrosis, metagenomics, whole-genome sequencing

## Abstract

Mutation-specific cystic fibrosis (CF) transmembrane conductance regulator (CFTR) modulator therapy with elexacaftor/tezacaftor/ivacaftor (ETI) has dramatically improved clinical outcomes for people with CF (pwCF), yet its impact on the nasal microbial metagenome remains insufficiently understood. This prospective, post-approval study investigated the impact of 15-week ETI therapy on sinonasal microbiota of pwCF aged 12 years and older. Whole-genome shotgun sequencing was performed on total DNA from 24 paired nasal lavage samples, with synthetic spike-in controls enabling absolute abundance normalization. Taxonomic profiling was conducted using the Wochenende pipeline. ETI did not induce major shifts in alpha or beta diversity. Instead, the overall microbial community became further dominated by the skin commensals *Staphylococcus epidermidis* and *Cutibacterium acnes*, accompanied by a more than twofold increase in total bacterial load. Classical CF pathogens showed divergent trajectories: *Pseudomonas aeruginosa* tended to decrease, whereas *Staphylococcus aureus* exhibited a tendency toward increased abundance. Co-occurrence network analysis revealed a transition from a dense, multicomponent baseline network to a single, fully connected, but less densely integrated network following treatment initiation.

**IMPORTANCE:**

The nasal cavity represents the primary entry point of microorganisms into the respiratory tract and a potential reservoir for lower airway infection, the major cause of CF disease progression. Using shotgun metagenomics with spike-in controls, this study provides the first genome-wide characterization of how ETI alters microbial load and pathogen dynamics in CF nasal airways. Treatment with ETI strengthened the dominance of skin commensals in the nares while reducing *P. aeruginosa*. Given the observed increase in *S. aureus*, further work is needed to determine whether this represents expansion of a typical nasal colonizer or a clinically relevant rise of a key CF pathogen that could act as a reservoir for future lower airway infection.

## INTRODUCTION

Cystic fibrosis (CF) is an autosomal-recessive disorder caused by mutations in the cystic fibrosis transmembrane conductance regulator (CFTR) gene, encoding a chloride and bicarbonate channel. CFTR is widely expressed in epithelial tissues, where its dysfunction impairs mucus hydration and viscoelasticity, leading to obstruction and sterile inflammation (1–3). In the lower airways, bacterial dysbiosis and chronic infection with hallmark pathogens *Staphylococcus aureus* and *Pseudomonas aeruginosa* drive persistent inflammation, mucus dysfunction, and progressive lung damage (4, 5). Although lung disease remains the main driver of mortality, upper-airway involvement also contributes substantial morbidity through chronic rhinosinusitis and nasal polyposis (6).

Similar to the lungs, impaired mucus clearance in the nose and sinuses promotes inflammation and chronic infection with hallmark pathogens *S. aureus* and *P. aeruginosa* (7–9), suggesting bidirectional colonization between the upper and lower airways and the establishment of a self-perpetuating cycle that is difficult to disrupt. Using shotgun metagenomics of nasal lavages, we recently showed that the nasal microbiome of people with CF (pwCF) is largely dominated by skin commensals, particularly *Staphylococcus epidermidis* and *Cutibacterium acnes* (10). Similar to others, we demonstrated that *S. aureus* frequently colonizes the nose of healthy subjects as well (10–19).

Over the past two decades, new therapeutic approaches improving CFTR function, termed CFTR modulators, have led to unprecedented improvements of CF lung disease, but also many other organ manifestations (20–28). The triple CFTR modulator combination therapy elexacaftor/tezacaftor/ivacaftor (ETI) potentially targets >90% of CF-causing mutations. Regarding lower airways, ETI has been shown to improve structural changes (29, 30), sputum viscoelasticity (3, 31), and to alter but not normalize microbial colonization with hallmark pathogens in adolescents and adult pwCF (32, 33). Upper-airway improvements, including sinus structural changes, have also been reported (34). Publications resorting to endoscopically sampled sinus material provide valuable information on the effect of ETI on the nasal microbiome. In these studies, surgically obtained samples were analyzed via 16S rRNA gene sequencing (35–38), complemented by custom-amplicon sequencing of *Staphylococcus* spp. and *Pseudomonas* spp. as CF hallmark pathogens (36, 37).

Similar to lower airway findings, these data show that ETI alters but fails to normalize sinus flora. Armbruster et al. (36) and Hilliam et al. (37) reported persistence of hallmark pathogens and chronic colonization with distinct *P. aeruginosa* clones in most pwCF after ETI. However, these cohorts were limited to adults requiring sinus surgery, many of whom had long-standing *P. aeruginosa* colonization, likely introducing a selection bias toward more severe nasal disease not representative of younger or less affected pwCF. In addition, samples were collected over broad pre- and post-ETI time windows, limiting temporal resolution and making it difficult to disentangle treatment effects from disease progression unrelated to ETI.

Conversely, Zemke et al. (35) and Stapleton et al. (38) studied younger cohorts with sampling performed immediately prior to ETI initiation. Notably, Zemke et al. (35) reported a marked reduction in *P. aeruginosa* abundance following ETI, with ecfX qPCR, utilized for the specific quantification of *P. aeruginosa*, falling below detection limits in all participants. However, by way of 16S sequencing, all these studies (35–38) fail to provide a comprehensive analysis of the entire microbiome. Metagenomic analysis of nasal lavages from unselected adolescents and adults with CF may extend these findings and address key clinical questions, including the extent of nasal microbial dysbiosis in CF, the efficacy of ETI to address nasal disease caused by microbial dysbiosis in CF, and the potential of ETI to reduce lower airway colonization through continuous exposure from chronic colonization of the upper airways.

In this study, we analyzed 24 paired nasal lavage samples from pwCF aged ≥12 years before and after 3 months of ETI using shotgun metagenomic sequencing. Our aim was to comprehensively characterize the impact of ETI on the nasal microbiome by assessing changes in microbial diversity, absolute and relative abundance, pathogen dynamics, and co-occurrence network structure in the upper airways of pwCF.

## RESULTS

### Study population characteristics and clinical outcomes

In this study, 29 people with CF (pwCF) were recruited and provided informed consent. Due to missing baseline (*n* = 2) or V1 samples (*n* = 1), missing clinical data (*n* = 2), and insufficient sequin counts in one sample, data from 24 individuals were included in the final analysis. The median age was 16.6 years (range: 12.1 to 44.8 years), and 13 of 24 participants were female (Table 1). Eight pwCF were homozygous for Phe508del, while 16 were compound heterozygous for Phe508del and a minimal-function mutation. Four participants had received prior CFTR modulator therapy, including lumacaftor/ivacaftor (LUM/IVA; *n* = 3) and tezacaftor/ivacaftor (TEZ/IVA; *n* = 1). The median interval between baseline and V1 was 109 days.

**TABLE 1:** Clinical, functional, and microbial parameters of CF patients at baseline and V1^*a*^^,^^*b*^

Study parameters		Baseline (median [range] or *n* [%])	V1 (median [range] or *n* [%])	mean difference	95% CI	*P* value
No. of patients (*n*)		24	24			
Age (years)		16.60 (12.10 to 44.81)				
Sex (female)		13 (54 %)				
Phe508del homozygous		8 (33 %)				
Phe508del heterozygous		16 (67 %)				
CFTR-modulator pre-treatment		4 (17 %)				
LUM/IVA		3 (13 %)				
TEZ/IVA		1 (4 %)				
Time period baseline to V1 (days)		109 (46 to 148)				
Clinical parameters						
FEV1 (%)		84.76 (47.80 to 117.32)	97.82 (59.19 to 132.43)			
∆ FEV1 (%) to baseline			+14.71 (−0.48 to 30.72)	15.35	11.27; 19.34	<0.001
MEF25 (%)		51 (13 to 99)	91 (22 to 211)			
∆ MEF25 (%) to baseline			+26 (−25 to 140)	31.58	16.51; 46.66	<0.001
LCI (CEV/FRC)		11.245 (8.06 to 21.57)	8.465 (7.26 to 18.72)			
∆ LCI to baseline			−2.215 (−5.34 to 1.25)	−2.061	−3.11; −1.01	<0.001
Sweat chloride (mmol/L)		103.5 (75 to 115)	45.5 (14 to 90)			
∆ Sweat chloride (mmol/L) to baseline			−52.5 (−89 to −18)	−53.25	−61.63; −44.87	<0.001
Leucocytes (thous./µL)		7.8 (5.0 to 10.5)	6.2 (4.8 to 11.9)			
∆ Leucocytes (thous./µL) to baseline			−0.95 (−3.4 to 3.1)	−0.64	−1.50; 0.21	0.132
BMI percentile#		29.4 (0.5 to 81.6)	36.4 (4.5 to 90.3)			
∆ BMI percentile to baseline			+5.5 (−15.5 to 26.2)	5.83	1.73; 9.92	0.007
Metagenome diversity parameters (all samples)						
Species richness (*n*)		4.5 (2 to 42)	7 (2 to 44)			
Pielou's evenness index		0.76 (0.08 to 1.00)	0.65 (0.18 to 0.94)			
Shannon diversity index		1.02 (0.09 to 2.95)	1.19 (0.20 to 2.98)			
Simpson diversity index		0.57 (0.03 to 0.92)	0.61 (0.08 to 0.92)			
Bacterial DNA load						
Total		0.64 (0.04 to 10.10)	1.66 (0.05 to 11.07)			
*P.aeruginosa* (in baseline/V1-positive patients)		0.04 (0.003 to 0.676)	0.00 (0.00 to 0.17)			
*H. influenzae* (in baseline/V1-positive patients)		0.10 (0.00 to 1.09)	0.00 (0.00 to 4.95)			
*S. aureus* (in baseline/V1-positive patients)		0.00 (0.00 to 0.14)	0.05 (0.00 to 2.98)			

^*a*^ LUM/IVA, lumacaftor/ivacaftor; TEZ/IVA, tezacaftor/ivacaftor; FEV1, forced expiratory volume in 1 second capacity in percent predicted; MEF25, mid expiratory flow at 25% of vital capacity in percent predicted; LCI, lung clearance index; BMI, body mass index.

^*b*^ The bacterial DNA load is based on sequin-normalized data, calculated as the ratio of RPMM values (number of reads per million base pairs of reference genome and million sequencing reads) assigned to each species to the RPMM values of the spiked-in internal reference standard (sequins). Age-adjusted BMI percentiles were obtained as stated in the supplementary material. Detailed clinical, functional, and microbial comparisons of the recruited (*n* = 29), included (*n* = 24), and CFTR modulator–treatment–naive (*n* = 20) participants at baseline and V1 are presented in Table S2.

Consistent with outcomes reported in pivotal CFTR modulator trials (26, 39), significant improvements in forced expiratory volume in 1 s (FEV_1_), mid-expiratory flow at 25% (MEF_25_), lung clearance index (LCI), sweat chloride concentration, and body mass index (BMI) were observed following ETI initiation, alongside a trend toward reduced serum leukocyte counts (Table 1).

### Biodiversity dynamics during ETI treatment

**Fig 1:**
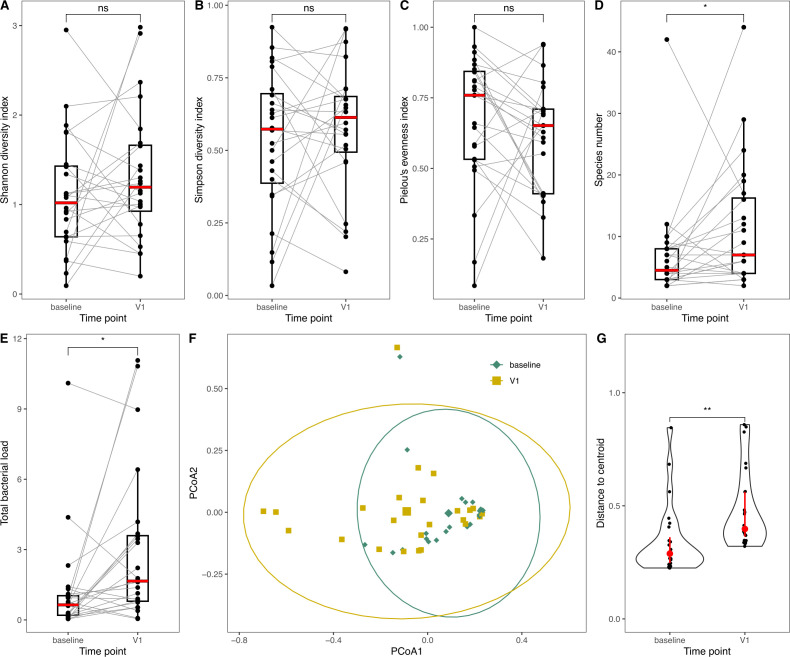
(**A–E**) Pairwise comparison of microbiome parameters between baseline and first study visit (V1) samples. (**A**) Shannon diversity (Wilcoxon adjusted *P* value [*P*] = 0.449, *r* = 0.157, confidence interval [CI] −0.1790–0.5196). (**B**) Simpson diversity (*P* = 0.9658, *r* = 0.0117, CI = −0.0916–0.1538). Gini-Simpson index is displayed. (**C**) Pielou’s evenness (*P* = 0.1573, *r* = 0.292, CI = −0.1861–0.0678). (**D**) Species richness (*P* = 0.0237, *r* = 0.486, CI = 0.5001–8.499). (**E**) Total bacterial load (*P* = 0.0146, *r* = 0.502, CI = 0.2252–2.5618). Absolute abundance values are based on sequin-normalized data, calculated as the ratio of RPMM values (number of reads per million base pairs of reference genome and million sequencing reads) assigned to each species to the RPMM values of the spiked-in internal reference standard (sequins). (**F**) Principal coordinate analysis (PCoA) based on a Euclidean-distance matrix derived from log10-transformed absolute microbial abundance values in each nasal lavage sample. Larger shapes indicate the centroids of each group. (**G**) To assess the multivariate homogeneity of variances between baseline and first study visit (V1) samples, the distance of each data point from its group centroid was calculated, and the data spread was compared across the time points. *P* = 0.0045, *r* = 0.584, CI = 0.0418–0.2271. ns, not significant/ *P* > 0.05; *, *P* < 0.05; **, *P* < 0.01.

While alpha diversity, as evaluated by Shannon and Simpson diversity indices and Pielou’s evenness index, remained unchanged (all *P* > 0.05, Wilcoxon signed-rank test), significant increases were observed in species richness (*P* = 0.0237, median at baseline = 4.5, median at V1 = 7, Wilcoxon signed-rank test) and total bacterial load (*P* = 0.0063, median at baseline = 0.64, median at V1 = 1.66, Wilcoxon signed-rank test) following ETI initiation (Fig. 1A through E). Apart from spike-in DNA-based normalization using synthetic sequins (40), an alternative approach for estimating absolute abundance (41, 42) yielded comparable results, showing a significant increase in bacterial cells per human cell after ETI initiation (*P* < 0.05; median at baseline = 2.12; median at V1 = 4.04; Wilcoxon signed-rank test).

**Fig 2:**
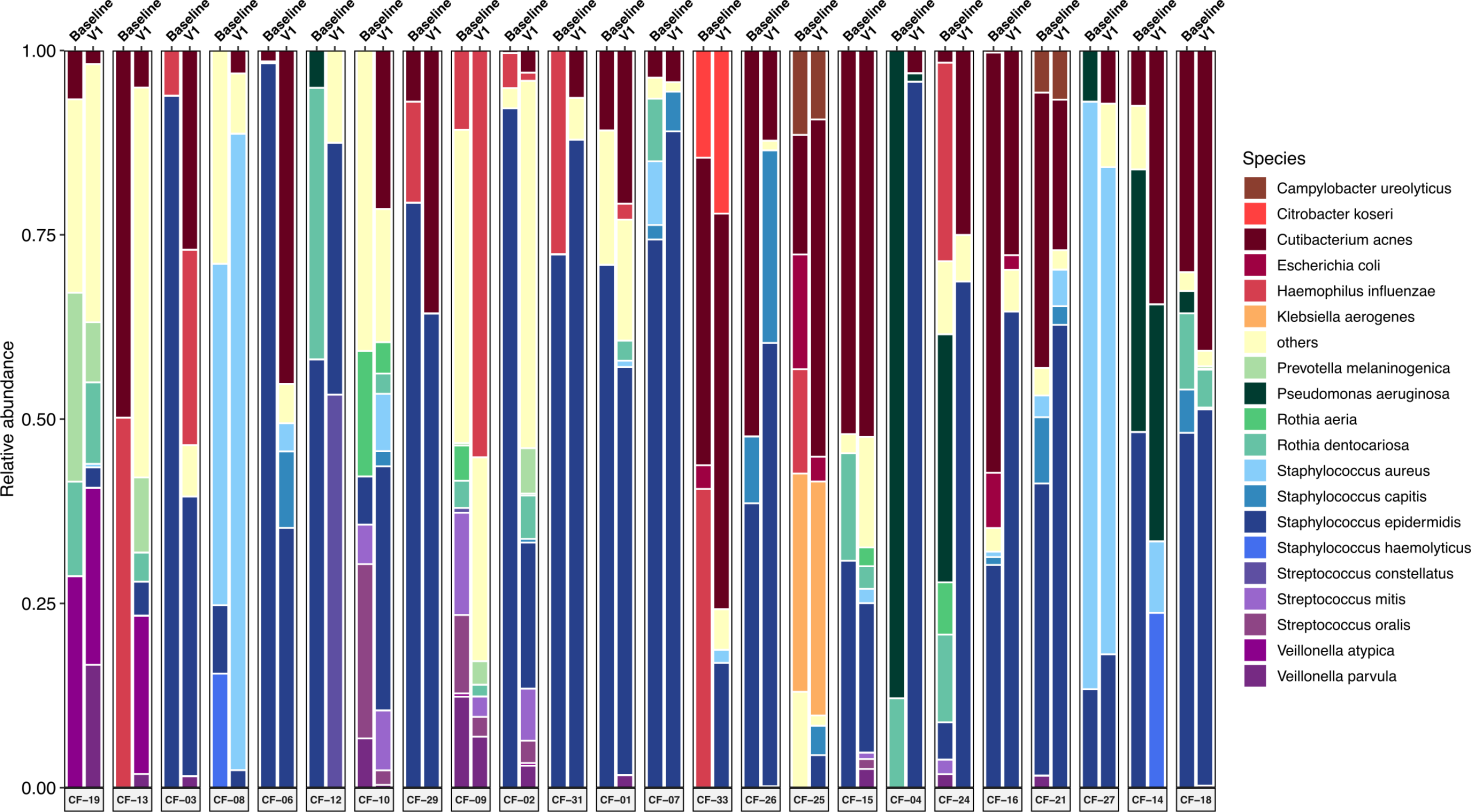
Overview of core and rare species relative abundances in CF airway metagenomes at baseline and after 15 weeks (V1) of ETI treatment. The bars and legend are sorted alphabetically. The colors represent taxonomic classification at the species level. Baseline and V1 samples of one patient are grouped together and labeled with the patient’s pseudonym (CF-XX). Samples are ordered by age of study participants from left to right. Rare species are defined as those outside the 19 most abundant species, which together accounted for 90% of the total microbial abundance across all samples, and are summarized as “others” (Table S5). Absolute species abundance values are based on sequin-normalized data, calculated as the ratio of RPMM values (number of reads per million base pairs of reference genome and million sequencing reads) assigned to each species to the RPMM values of the spiked-in internal reference standard (sequins), and subsequently were converted to relative values.

**Fig 3:**
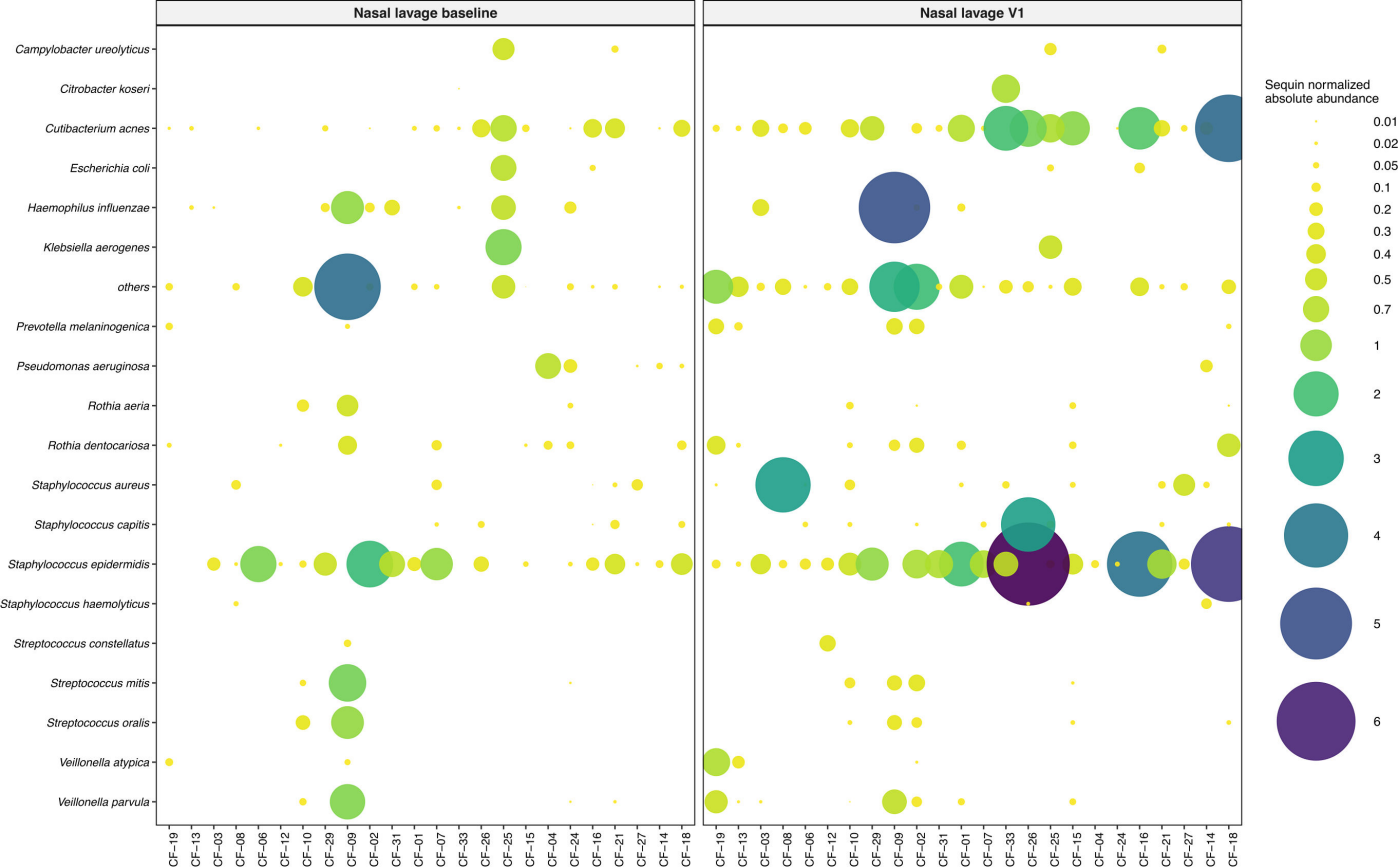
Overview of core and rare species absolute abundances in CF airway metagenomes at baseline and after 15 weeks (V1) of ETI treatment. Samples are ordered by time point (baseline, V1) and age and labeled with patients’ pseudonyms. Rare species are defined as those outside the 19 most abundant species, which together accounted for 90% of the total microbial abundance across all samples, and are summarized as “others” (Table S5). Absolute species abundance values are based on sequin-normalized data, calculated as the ratio of RPMM values (number of reads per million base pairs of reference genome and million sequencing reads) assigned to each species to the RPMM values of the spiked-in internal reference standard (sequins).

**Fig 4:**
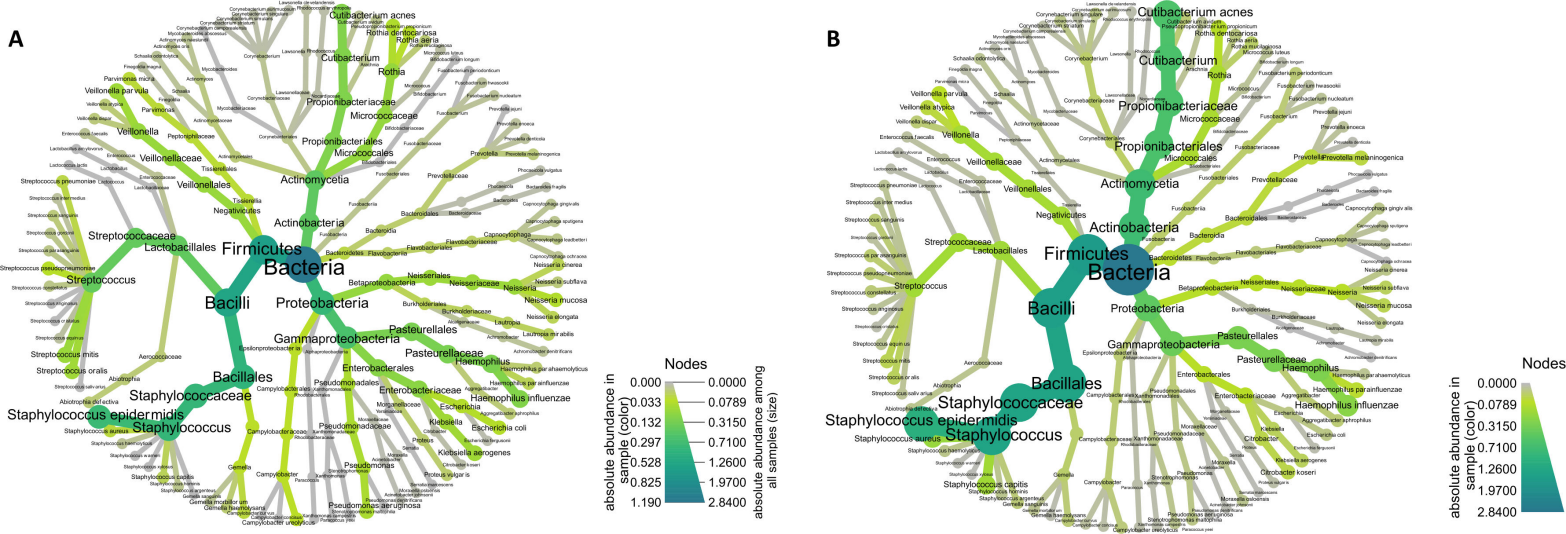
Heat tree representation of mean absolute species abundances at baseline (**A**) and after 15 weeks of treatment with ETI (V1) (**B**) across all samples. Absolute abundance values are based on sequin-normalized data, calculated as the ratio of RPMM values (number of reads per million base pairs of reference genome and million sequencing reads) assigned to each species to the RPMM values of the spiked-in internal reference standard (sequins). Heat trees were generated by the Wochenende pipeline. Both heat trees include all species, whether detected at baseline or V1, for optimized comparability. (**A**) Absolute abundance at baseline is indicated by color, while combined absolute abundance across both time points (baseline and V1) is represented by the size of the species names. (**B**) Absolute abundance at V1 is represented by both the color and size of the species names.

The increase in total bacterial load was primarily driven by *S. epidermidis* and *C. acnes*, typical skin colonizers (43) (Fig. 2 to 4). While many taxa showed a tendency toward increased abundance, this pattern was not consistent across all species or patients. In particular, *Pseudomonas aeruginosa* exhibited an overall decrease in abundance, whereas *Haemophilus influenzae* showed an apparent increase driven by individual outliers, despite a general tendency toward decreased abundance among most patients (Fig. 3 and 6).

We assessed beta diversity by conducting principal coordinate analysis based on Euclidean distance matrices to evaluate the impact of clinical and microbial parameters on the core nasal microbial community structure. Beyond *P. aeruginosa* and *Prevotella* spp., sex, patient variability, and the time point (baseline vs V1) significantly influenced the core microbial composition (Table 2). Nevertheless, analysis uncovered substantial overlap in nasal microbial community profiles before and during ETI treatment (Fig. 1F). Notably, V1 samples showed significantly greater dispersion around the group centroid than baseline samples (*P* < 0.01) (Fig. 1G), indicating increased interindividual variability and the emergence of individualized nasal metagenomic signatures under ETI.

**TABLE 2:** Principal coordinate analysis (PCoA) based on a Euclidean-distance matrix^*a*^^,^^*c*^

Parameters	Goodness of fit, *r*^2^	Goodness of fit, *P*
Clinical parameters		
Patient	0.6303	0.037^*b*^
Time point	0.1016	0.012^*b*^
Sex	0.0663	0.040^*b*^
Genotype	0.0343	0.196
Pre-treatment	0.0001	0.999
Pathogens		
*P. aeruginosa*	0.0805	0.028^*b*^
*H. influenzae*	0.0371	0.175
*S. aureus*	0.0038	0.857
Commensals		
Overall healthy representatives	0.0161	0.475
*Streptococcus* spp.	0.0576	0.059
*Rothia* spp.	0.0353	0.186
*Veillonella* spp.	0.0430	0.131
*Neisseria* spp.	0.0457	0.128
*Prevotella* spp.	0.0949	0.016^*b*^

^*a*^ The significance of known factors fitted to the ordination was determined using a permutation test (*n *= 999, R vegan package, envfit).

^*b*^ *P* < 0.05.

^*c*^ Overall healthy representatives comprise a combination of *Streptococcus *spp., *Prevotella *spp., *Rothia *spp., *Neisseria *spp., and *Veillonella *spp.

### Impact of ETI on species co-occurrence networks

To evaluate the effects of ETI on species interaction patterns, ecological community dynamics, and microbial cohabitation structure, species co-occurrence network analyses were performed. These networks illustrate the interconnectedness of taxa based on their simultaneous presence or absence within a given habitat (44).

**Fig 5:**
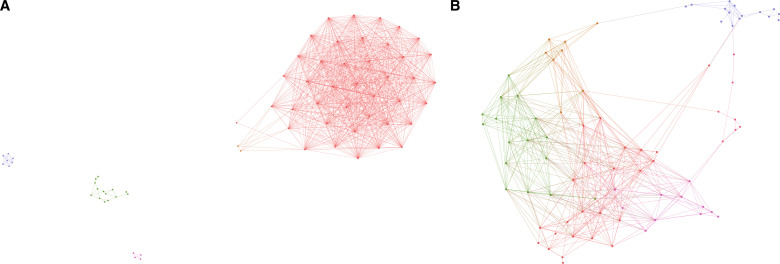
Evolution of species co-occurrence networks from CF nasal lavage samples at baseline and after 15 weeks (V1) of ETI. Taxa within each modularity class of the baseline and V1 co-occurrence networks are listed in Table S4. (**A**) Baseline network with five modularity classes: red (60.29%), green (20.59%), purple (10.29%), pink (5.88%), and yellow (2.94%), ordered by decreasing network contribution. (**B**) First study visit network with six modularity classes: red (29.63%), green (19.75%), purple (18.52%), pink (13.58%), yellow (9.88%), and rose (8.64%), ordered by decreasing network contribution.

At baseline, the network consisted of four distinct components, with the largest showing exceptionally high density and interconnectedness (Fig. 5A). After 15 weeks of ETI (V1), however, the overall network exhibited markedly reduced density and connectivity (Fig. 5B). Statistical metrics, including decreases in the number of interconnections, average degree, graph density, and the contribution of the largest network component to the overall network from baseline to V1 (Table S3), together with trajectories of Node authority, Betweenness centrality, and Closeness centrality (Fig. S1F through H), further supported this finding. In terms of the composition of the largest network component, the genera present (mainly *Neisseria* spp.*, Streptococcus* spp., *Rothia* spp., and *Gemella* spp.) were largely consistent from baseline to V1 (Fig. S1A), with only minor variations in alpha diversity and the number of genera (Fig. S1B through E).

Notably, the multicomponent network observed prior to treatment, which featured skin colonizers and *Enterobacterales* segregated from the main network (Fig. 5A, modularity classes green and purple; Table S4), with *P. aeruginosa* and *S. epidermidis* absent, evolved into a monocomponent network at V1. In this unified network, all species integrated into the core structure, with *P. aeruginosa* and *S. epidermidis* showing co-occurrence with other species for the first time. In parallel, the total number of integrated taxa increased from 68 to 81 (Table S3), indicating overall expansion of the network’s taxonomic breadth.

### Relative and absolute abundances of core and rare species in the CF nasal airway metagenome

Nasal lavage sampling of the upper airways revealed a predominant presence of the two skin colonizers *C. acnes* and *S. epidermidis*. In 60% of patient samples, these species collectively accounted for more than 50% of the total microbial abundance, and in half of the samples, their combined abundance exceeded 70% (Fig. 2 to 4). Only one patient (CF-09) lacked detection of either species at both baseline and V1. *S. epidermidis* was detected in 23 of 24 pwCF. Specifically, 17 participants were positive at both baseline and V1, five patients exhibited detection only at baseline, and one patient demonstrated detection exclusively at V1. *C. acnes* was detected in 21 out of 24 individuals. Of these, 16 were positive at both baseline and V1, while five patients exhibited positivity exclusively at V1.

**Fig 6:**
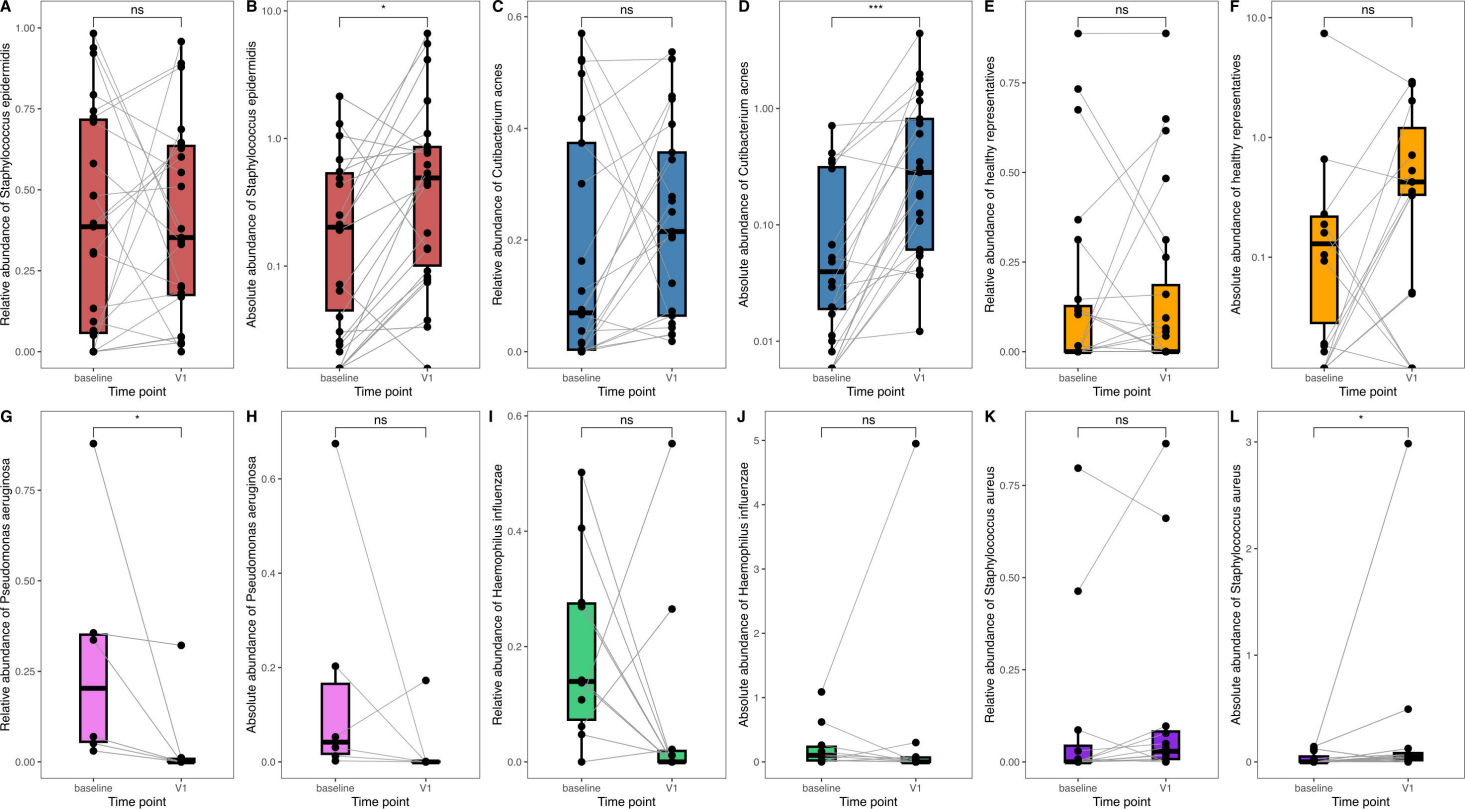
Pairwise comparisons of relative (**A, C, E, G, I, and K**) and absolute (**B, D, F, H, J, and L**) abundances between baseline and first study visit (**V1**) samples in CF patients. Analyses were restricted to species-positive patients. (**A and B**) *Staphylococcus epidermidis*: (**A**) relative abundance, Wilcoxon adjusted *P* value [*P*] = 0.8345, *r* = 0.069, confidence interval (CI) = −0.1919–0.2029; (**B**) absolute abundance, *P* = 0.0101, *r* = 0.54, CI = 0.0645–0.959, y-axis is scaled logarithmically. (**C and D**) *Cutibacterium acnes*: (**C**) relative abundance, *P* = 0.1443, r = 0.323; CI = −0.0487–0.1674; (**D**) absolute abundance, *P* = 0.0003, *r* = 0.777, CI = 0.1007–0.9764; y-axis is scaled logarithmically. (**E and F**) Representative genera of a healthy nasal microbiome (*Streptococcus* spp.*, Prevotella* spp.*, Rothia* spp.*, Neisseria* spp.*,* and *Veillonella* spp.): (**E**) relative abundance, *P* = 1, *r* = 0, CI = −0.175–0.1712; (**F**) absolute abundance, *P* = 0.222, *r* = 0.323, CI = −0.1261–0.9308; y-axis is scaled logarithmically. (**G and H**) *Pseudomonas aeruginosa*: (**G**) relative abundance, *P* = 0.036, *r* = 0.898, CI = −0.4686–0.0344; (**H**) absolute abundance, *P* = 0.2084, *r* = 0.555, CI = −0.3523–0.0535. (**I and J**) *Haemophilus influenzae*: (**I**) relative abundance, *P* = 0.2213, *r* = 0.402, CI = −0.3218–0.1126; (**J**) absolute abundance, *P* = 0.6103, *r* = 0.177, CI = −0.2713–1.8501. (**K and L**) *Staphylococcus aureus*: (**K**) relative abundance, *P* = 0.1955, *r* = 0.384, CI = −0.0342–0.0874; (**L**) absolute abundance, *P* = 0.0254, *r* = 0.655, CI = 0.0131–0.2381. Note: Absolute abundance measurements are based on sequin-normalized data, calculated as the ratio of RPMM values (number of reads per million base pairs of reference genome and million sequencing reads) assigned to each species to the RPMM values of the spiked-in internal reference standard (sequins). ns, not significant. *P* > 0.05; *, *P* < 0.05; **, *P* < 0.01; ***, *P* < 0.001.

Following ETI initiation, relative abundances of *S. epidermidis* and *C. acnes* did not change significantly (both *P* > 0.05, Wilcoxon signed-rank test), but absolute abundances of both *S. epidermidis* (*P* = 0.0101, median at baseline = 0.07, median at V1 = 0.45, Wilcoxon signed-rank test) and *C. acnes* (*P* = 0.0003, median at baseline = 0.02, median at V1 = 0.28, Wilcoxon signed-rank test) increased significantly under ETI (Fig. 4 and 6A through D).

Subgroup analyses of rare species (Table S5), defined as those outside the 19 species with the highest mean relative abundance across all samples (together accounting for 90% of total abundance), revealed a similar pattern (Fig. 2 and 3): their relative abundance remained unchanged (*P* > 0.05, Wilcoxon signed-rank test), whereas their absolute abundance increased significantly following ETI (*P* = 0.0408, median at baseline = 0.02, median at V1 = 0.11, Wilcoxon signed-rank test).

Bacteria associated with a healthy microbiome within the nasal cavity and beyond—apart from the skin colonizers *S. epidermidis* and *C. acnes*—including *Neisseria* spp., *Prevotella* spp., *Rothia* spp., *Streptococcus* spp., and *Veillonella* spp. (10, 32, 45, 46), remained largely unaffected in both relative and absolute abundance (both *P* > 0.05, Wilcoxon signed-rank test) following ETI (Fig. 6E and F).

### Effects of ETI on the abundance of common CF pathogens

Overall, in all six patients positive for *P. aeruginosa* at baseline, the relative abundance of *P. aeruginosa* decreased significantly (*P* = 0.036, median at baseline = 0.2, median at V1 = 0, Wilcoxon signed-rank test) (Fig. 6G), but reduction in absolute abundance was not statistically significant (*P* > 0.05, Wilcoxon signed-rank test) (Fig. 6H). Six patients exhibited *P. aeruginosa* in sequencing prior to ETI (CF-04, CF-12, CF-14, CF-18, CF-24, and CF-27), with four showing no detectable *P. aeruginosa* following treatment (CF-12, CF-18, CF-24, and CF-27) (Fig. 2, 6G and H). Among the two patients remaining positive at V1 (CF-04 and CF-14), the relative abundance of *P. aeruginosa* declined from 87.9% at baseline to 1.2% at V1 (CF-04) and from 35.6% at baseline to 32.2% at V1 (CF-14) (Fig. 4 and 6G). The only patient with increased absolute *P. aeruginosa* abundance following ETI initiation (0.05 at baseline to 0.17 at V1, CF-14) (Fig. 6H) was a 44-year-old proband who showed limited response in both pulmonary function (FEV1, +5.21%; MEF25, ± 0%) and nasal microbiome diversity (Shannon index, +0.17; Simpson index, +0.09; Pielou’s evenness, +0.13; species richness, ±0), as further corroborated by absolute abundance heat tree comparison between baseline and V1 (Fig. S2E and F).

Regarding *S. aureus*, relative abundance showed a nonsignificant upward trend (*P* > 0.05, Wilcoxon signed-rank test) (Fig. 6K), whereas absolute abundance increased significantly under treatment (*P* = 0.0254; median at baseline = 0.0, median at V1 = 0.05; Wilcoxon signed-rank test) (Fig. 6L). Of the 12 probands with *S. aureus* identified, two were positive before, but not during ETI treatment (CF-07, CF-16); three remained positive from baseline to V1 (CF-08, CF-21, CF-27); and seven patients, initially negative for *S. aureus*, became positive during ETI therapy (CF-01, CF-06, CF-10, CF-14, CF-15, CF-19, and CF-33) (Fig. 2, 6K and L). In summary, nine out of 24 pwCF exhibited elevated absolute abundance of *S. aureus* under ETI.

Regarding *H. influenzae*, neither relative nor absolute abundance decreased significantly following ETI (both *P* > 0.05, Wilcoxon signed-rank test) (Fig. 6I and J). Among the 10 probands with *H. influenzae* detected, six tested positive before but not during ETI treatment (CF13, CF-24, CF-25, CF-29, CF-31, and CF-33); three remained positive from baseline to V1 (CF-02, CF-03, and CF-09); and one patient, initially *H. influenzae*-negative, became positive under ETI therapy (CF-01) (Fig. 2, 6I and J). Two pwCF (CF-03 and CF-09) exhibited an increase in both relative and absolute abundance of *H. influenzae*. Although not statistically significant, seven of 10 pwCF with detectable *H. influenzae* showed a decline under ETI, with six becoming negative at V1.

### Associations between metagenomic variables and clinical parameters

Spearman’s rank-order correlations were performed separately for baseline and V1 data sets to evaluate relationships among metagenome parameters, as well as between clinical and metagenome parameters, including demographic patient characteristics, pulmonary function, sweat chloride concentration, and inflammatory indicators.

**Fig 7:**
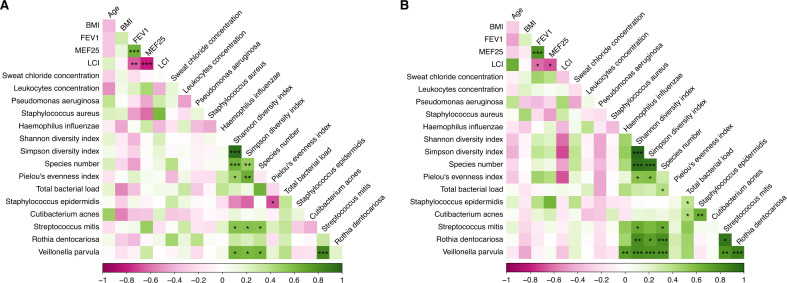
Spearman’s rank-order correlations of clinical and microbial metagenome parameters at baseline (**A**) and 15 weeks of treatment with ETI (V1) (**B**). Spearman’s rank-order correlations were obtained between patient characteristics (age, BMI), pulmonary function (FEV1, MEF25, and LCI), quantitative CFTR biomarkers (sweat chloride concentration), inflammation parameters (leukocyte concentration), culture-independent abundance of common CF pathogens (*P. aeruginosa*, *S. aureus*, and *H. influenzae*), and metagenome and alpha diversity parameters, including Shannon diversity index, Simpson diversity index, species number, Pielou’s evenness index, bacterial load, and abundance of *Staphylococcus* spp., *Cutibacterium* spp., *Streptococcus* spp., *Rothia* spp., and *Veillonella* spp. *P* values were adjusted for multiple testing by Benjamini-Hochberg correction. *, *P* < 0.05; **, *P* < 0.01; ***, *P* < 0.001.

Common CF pathogens showed predominantly solitary occurrence patterns that were stable from baseline to V1. In contrast, healthy airway microbiome members, including *Streptococcus mitis*, *Rothia dentocariosa*, and *Veillonella parvula*, showed synergistic interactions and significantly contributed to nasal microbiome diversity and species richness (Fig. 7). Specifically, at baseline, *S. mitis* and *V. parvula* significantly correlated positively with diversity indices and richness. At V1, these correlations strengthened for *V. parvula*, while *R. dentocariosa* also showed significant positive associations with diversity parameters. Furthermore, whereas only *V. parvula* significantly correlated with *S. mitis* at baseline, all three commensal species showed significant positive intercorrelations at V1.

*S. epidermidis* showed a significant negative correlation with Pielou’s evenness at baseline, but not at V1. In contrast, at V1, *S. epidermidis* and *C. acnes* were significantly correlated with total bacterial load and with each other, associations not observed at baseline.

Across both baseline and V1, Shannon diversity, Simpson diversity, Pielou’s evenness, and species richness were significantly intercorrelated, except for the association between Pielou’s evenness and species richness. Total bacterial load showed a significant positive correlation with species richness exclusively at V1. Interestingly, *V. parvula* significantly correlated with *H. influenzae* at V1, whereas no such association was observed at baseline.

Regarding patient characteristics, no significant correlations were identified at either baseline or V1 beyond expected lung function parameter relationships. Notably, correlations of LCI with FEV1 and MEF25 were weaker at V1 compared with baseline.

## DISCUSSION

This prospective, post-approval study assessed how ETI therapy influences the nasal microbial metagenome in pwCF. After 15 weeks of treatment, alpha and beta diversity remained largely unchanged; the microbial co-occurrence network shifted from a four-component, highly dense structure to a single, fully connected component with markedly lower density and connectivity; the dominance of the typical skin colonizers *S. epidermidis* and *C. acnes* in the nasal microbiome was further amplified, accompanied by a more than twofold increase in overall bacterial load; and hallmark CF pathogens exhibited divergent trends, with *P. aeruginosa* tending to decrease, while *S. aureus* showed a tendency toward increased abundance. Our data complement previous studies on microbial composition and ETI-induced changes in upper airways, suggesting that airway responses of upper and lower airways are interconnected critically.

Studies of ETI effects on the lower airways using sputum generally report increased alpha and beta diversity after treatment (3, 33, 47, 48). In contrast, we recently found no significant changes in Shannon, Simpson, or beta diversity, but observed increased Pielou’s evenness and reduced total bacterial load in induced sputum and deep-cough swabs from a cohort largely congruent with the present one (32). For the upper airways, previous studies have shown that alpha diversity in sinus samples is low and often reduced compared with paired sputum samples (49, 50). Consistent with Zemke et al. (35), who analyzed endoscopically obtained sinonasal swabs, we detected no significant changes in alpha or beta diversity in nasal lavage samples, apart from increased species richness. Nevertheless, nasal lavages collected at V1 exhibited significantly greater distances to their group centroid in multivariate space than pre-treatment samples, indicating a trend toward the development of individualized nasal metagenomic signatures under ETI therapy. In contrast, Hilliam et al. (37) observed variation in the beta diversity of sinus samples both within and between individuals, arguing that conditions related to chronic rhinosinusitis (CRS) and prior endoscopic sinus surgery (FEES) in their cohort may have affected microbial composition. Notably, the microbiome of nasal lavage samples has been shown to exhibit significantly higher alpha diversity and evenness than that of nasal swab samples in pwCF aged 7 to 19 years (51), supporting the use of nasal lavage as a promising tool for simultaneous assessment of the upper airway microbiome.

By shotgun metagenomic analysis of nasal lavage samples, we recently showed that the nasal microbiome of pwCF is largely dominated by *S. epidermidis* and *C. acnes* (10), typical skin commensals (43). The present study confirms this, as in 60% of all samples these species accounted for more than half of the total microbial abundance, and only one patient lacked either species at both baseline and V1. Although their relative abundances did not increase significantly, their absolute abundances did, further strengthening their dominance under ETI. 16S-based studies of nasal swabs likewise reported a *Staphylococcus*-dominated microbiome before and after ETI (35, 37). Zemke et al. (35) found a median relative abundance of approximately 60% with no significant treatment effect, while Hilliam et al. (37) observed sustained or increased relative abundance, with an average rise of 18.2%. However, because 16S amplicon sequencing cannot resolve species-level composition, these increases may reflect expansion of either commensal (e.g., *S. epidermidis*) or pathogenic (e.g., *S. aureus*) taxa. Indeed, using custom oligos, Hilliam et al. (37) showed that *S. aureus* and *MRSA*, alongside *S. epidermidis*, contributed substantially to *Staphylococcus* detection.

In line with these observations, our data indicate a shift in *S. aureus* dynamics under ETI. Relative abundance showed only a nonsignificant upward trend, but absolute abundance increased significantly, and most of the 12 *S. aureus*-positive individuals either remained positive or newly acquired the pathogen during therapy. Despite this increase, no significant differences were observed in lung function, BMI, or sweat chloride between *S. aureus*-positive and -negative individuals (all *P* > 0.05, Wilcoxon signed-rank test). This lack of association also persisted when comparing individuals with increased *S. aureus* levels to those with declining or persistently negative status. In the lower airways, most studies report reduced *S. aureus* detection in sputum or throat cultures under ETI (33, 52–56), consistent with 16S-based (33) and shotgun metagenomic findings (32, 57), although some report no reduction (47, 48) or even increases (58). Because changes in key CF pathogens might be indicative of CFTR modulator efficacy (32, 59–62), and the sinuses serve as reservoirs for lower-airway infection (7–10, 63), the impact of ETI on the sinonasal pathogen profile is crucial. Although *S. aureus* frequently colonizes healthy nares (11–19), its role as an early, prevalent CF airway pathogen (64) and major driver of lower-airway inflammation and damage (4, 5) highlights the need for careful monitoring of its increased presence in the nasal microbiome under ETI.

Regarding the lower airways, most studies report reduced *P. aeruginosa* detection in sputum or throat swab cultures after ETI initiation (33, 52, 54–56, 65, 66), with long-term declines in culture density observed up to 3.5 years after treatment onset (56). Using shotgun metagenomics, we recently observed significant decreases in *P. aeruginosa* load in sputum and throat swabs after 3 months and 1 year of therapy (32), consistent with 16S-based reports of decreased relative abundance (3, 33), although some studies found no decline (47, 48). For the upper airways, 16S sequencing of endoscopically obtained nasal swabs likewise demonstrated reduced relative abundances of *P. aeruginosa* under ETI (35–37). However, distinct clones persisted in most pwCF despite treatment (36, 37), indicating a lack of eradication. In our study cohort, six patients were positive for *P. aeruginosa* at baseline, four of whom became undetectable at V1. Relative abundance decreased significantly, whereas the decline in absolute abundance did not reach significance. Only one individual exhibited increased absolute abundance, and this patient also showed a limited clinical and microbiome response to ETI, possibly reflecting advanced disease and chronic *P. aeruginosa* colonization (Fig. S2E and F). Collectively, these findings indicate a substantial ETI-associated decline of *P. aeruginosa* in the upper airways, while also demonstrating that persistence may occur in individuals with limited therapeutic response, whereas eradication appears achievable in responders.

Regarding *H. influenzae*, findings across studies are mixed. Steinberg et al. (57) reported reduced detection in adolescents using shotgun metagenomics, whereas Mianowski et al. observed increased detection in sputum cultures after ETI initiation (54). In our recent shotgun metagenomic analysis of sputum and throat swabs, *H. influenzae* load decreased significantly at 3 months and 1 year after ETI onset, although six patients showed new detection during therapy (32). In the present cohort, neither relative nor absolute abundance declined significantly with a mixed picture among individual participants. Of 10 participants with detectable *H. influenzae*, six converted from positive to negative, two showed increases in both relative and absolute abundance, and one patient converted from negative to positive. Numerical increases in absolute abundances were mainly driven by increases in one patient (CF-09), accompanied by a marked loss of nasal microbiome diversity and only modest improvement in lung function (Fig. S2C and D).

Overall, together with additional case reports (Fig. S2A, B, G and H), these findings support our hypothesis that improvements in the nasal microbiome toward a healthier state mirror the overall clinical response to modulator therapy (67, 68). Moreover, the pronounced interindividual variation in both clinical and microbial outcomes within this cohort (age range 12 to 44 years) is consistent with the widely accepted view that the age at ETI initiation, and thus the underlying severity of lung disease, is a major determinant of treatment response, indicating that not all pwCF will benefit equally from ETI (56, 69, 70).

A key contrasting finding of our study compared to previous studies is the significant, more than twofold increase in total bacterial load under ETI (median at baseline = 0.64; median at V1 = 1.66). Most 16S-based studies of sputum (3, 47, 48) and nasal swabs (35, 37) report no significant change in total bacterial load during ETI. Consistent with the findings of Armbruster et al. (36), we previously observed significant decreases in total bacterial load after one year of ETI in sputum and throat swabs (32). To our knowledge, an increase in total bacterial load under ETI has not been reported previously. To facilitate normalization and potentially improve reliability, absolute abundances were derived using synthetic spike-in controls (*sequins*), which are added in fixed amounts before library preparation and sequenced alongside sample DNA. Quantification of sequin reads enables conversion from relative to absolute abundances and supports comparison of total microbial load across samples (40). Moreover, normalization using the alternative metric of bacterial cells per human cell (41, 42) similarly indicated an increase in microbial absolute abundance under ETI, lending further support to this observation. The rise in total bacterial load was largely driven by an increased dominance of the skin commensals *S. epidermidis* and *C. acnes*, although most taxa, apart from the CF pathogen *P. aeruginosa*, showed a tendency toward higher abundances under ETI. Previously, we proposed that although *S. epidermidis* and *C. acnes* are not typical residents of healthy nasal conchae, the CFTR defect predisposes pwCF to chronic rhinosinusitis and nasal obstruction, likely promoting colonization by these skin commensals (10). Their increased abundance under ETI suggests that correcting the defect does not reduce these taxa; instead, the decline of classical CF pathogens may create a niche that these commensals especially readily occupy under ETI-modified conditions.

Previously, in sputum and throat swabs, we observed that a multicomponent co-occurrence network present before ETI initially transitioned into a more unified and interconnected structure after 3 months of therapy, but this configuration became unstable again after 1 year (32). In contrast, the nasal microbiome in the present study shifted from a highly interconnected four-component network at baseline to a unified but markedly less dense network at 3 months after ETI initiation. Despite this restructuring, the diversity and number of genera within the main modularity class remained largely stable, and the total number of integrated taxa increased. These altered interaction patterns may again reflect the concurrent rise of skin commensals and decline of the pathogen *P. aeruginosa* under ETI. Importantly, long-term dynamics remain uncertain, as our previous work suggests that early ETI-associated shifts in microbial community structure may not persist over time.

As expected, diversity measures and commensal abundance (32, 71–73), as well as spirometry (32, 39, 74), correlated within their respective functional domains (microbiome and lung function). However, the CF nasal microbiome showed a clear divide: classical CF pathogens had few associations with other taxa before and during ETI therapy, whereas health-associated commensals formed a tightly interconnected network that strongly contributed to diversity and richness, a pattern that became more pronounced after ETI initiation. In addition, skin commensals correlated with total bacterial load only at V1, supporting their contribution to the ETI-associated increase in absolute bacterial abundance, while their positive intercorrelations suggest potential reciprocal enhancement. Notably, the significant negative correlation between *S. epidermidis* abundance and Pielou’s evenness observed at baseline disappeared at V1, indicating that increased abundance under ETI did not promote community imbalance through *S. epidermidis* dominance. This is further supported by the positive correlation between total bacterial load and species richness observed only at V1, suggesting that increased bacterial abundance under ETI reflected broader species expansion rather than overgrowth of skin commensals alone.

This study has several limitations. First, microbial DNA was heavily outnumbered by host-derived DNA, which is typical for clinical specimens (75), reducing the proportion of microbial reads and potentially limiting detection of low-abundance taxa. Although sufficient sequencing depth and use of the Raspir tool (76) enabled detection of such taxa for downstream analyses, this imbalance remains an inherent constraint of our approach. Second, the sample size was relatively small (*n* = 24 paired nasal lavages), which may have limited detection of statistically significant changes in pathogen abundance. Third, ETI effects were assessed over only 15 weeks, limiting conclusions about the long-term stability and clinical relevance of nasal microbiota shifts, particularly given the reported instability of ETI-induced lower-airway changes over time (32). Fourth, although our cohort (12 to 44 years) broadly reflects the CF population, ETI is effective from 2 years of age (77, 78) and approved for younger children. Its impact on the nasal microbiome in this group remains to be clarified.

In summary, our post-approval study demonstrates that treatment with ETI reinforces the dominance of skin commensals as key residents in the nares while reducing the abundance of *P. aeruginosa* in CF nasal microbiota. Although these findings may suggest a shift toward a more health-associated nasal microbial composition, their biological and clinical implications remain uncertain. The observed reorganization of co-occurrence networks, from a dense, multicomponent structure at baseline to a less connected single-component network under ETI, indicates substantial ecological remodeling. However, whether this configuration represents a more stable or favorable microbial state is unclear, particularly given the lack of a well-defined reference for a healthy nasal microbiome in CF. Moreover, the trend toward increased *S. aureus* abundance underscores the need for cautious interpretation, as this species may represent either a common nasal commensal or a clinically relevant CF pathogen with potential implications for lower-airway infection. Longer-term longitudinal studies are required to determine the persistence, stability, and clinical relevance of ETI-associated changes in the upper-airway microbiome.

## MATERIALS AND METHODS

### Study design, participants, and clinical examinations

Eligible participants were pwCF aged 12 years or older who were either compound heterozygous for p.Phe508del and a minimal function mutation or homozygous for p.Phe508del. All participants were treatment-naïve to ETI before enrollment and agreed to maintain a stable drug regimen with ETI throughout the study. Exclusion criteria included acute respiratory infection or pulmonary exacerbation at baseline and a history of transplantation. Nasal lavage samples were collected at baseline and again at a median of 109 days (15 weeks) after initiating ETI (V1) at Hannover Medical School. Spirometry, anthropometric measurements, and sweat chloride concentration were assessed at both study visits as previously described (32).

### Nasal lavage sample collection

If necessary, patients were instructed to blow their nose prior to the procedure. A total of 10 mL of 0.9% NaCl was drawn into a syringe. Patients were asked to close the soft palate. With the head tilted to the side to be irrigated and slightly reclined, 10 mL of saline was instilled into the nostril and retained for approximately 10 s without swallowing. Patients then leaned forward, allowing the fluid to drain into a sterile plastic beaker while gently exhaling through the nose. The procedure was repeated for the opposite nostril. The uncentrifuged nasal effluent was transferred into a 15 mL Falcon tube and immediately stored at −80°C until further processing.

### Contamination prevention and quality assurance

To minimize DNA contamination, all equipment was cleaned with a 2% (wt/vol) sodium hypochlorite solution. Sample processing was conducted under aseptic conditions in a UV-PCR workstation, with personnel using disposable laboratory coats, sterile gloves, and mouth and hair coverings, as previously described (32, 73). Negative controls (empty water controls) were stored, processed, and sequenced alongside patient samples to ensure continuous quality control throughout the experiments (Fig. S4).

### DNA extraction and fragmentation

Frozen nasal lavage samples, stored in 15 mL Falcon tubes at −80°C, were thawed at room temperature. A 2 mL aliquot was transferred into a 2 mL Eppendorf tube and incubated in a 65°C heating block for 60 s. The sample volume was then reduced to 200 µL using a vacuum centrifuge (45°C, 20 mbar, 2,000 rpm, 402.48 × g; duration depended on number of processed samples). For cell lysis, 200 µL of nasal lavage was subjected to four freeze-thaw cycles, each consisting of 5 min on dry ice, followed by 3 min in a 65°C heating block. The lysate was transferred to a 1.5 mL Eppendorf tube, sealed with Parafilm, and processed using the S220 Focused-ultrasonicator (Covaris, program 1, Table S6). Next, 128.8 µL of the sample was transferred to a Covaris microTUBE, and 0.2 ng of sequencing spike-ins (sequins) (40) in a 1.2 µL solution was added, bringing the total volume to 130 µL. The tube was sealed with Parafilm. DNA fragmentation was performed using the Covaris system (program 2, Table S6), generating fragments approximately 250 base pairs in length. The fragmented DNA solution was transferred to 1.5 mL Eppendorf tubes and centrifuged (3 min, 13,000 × *g*, room temperature). For purification, the supernatant was mixed with 156 µL of AMPure XP Beads (A63881; Beckman Coulter) and incubated at room temperature for 4 min. The tube was then placed on a magnetic rack, and the clear supernatant was discarded. The pellet was washed three times with 80% ethanol, resuspended in 30 µL of TE buffer, and incubated (2 min, room temperature) before being placed on the magnetic rack. The clear eluate was transferred to a PCR tube for library preparation and stored at 4°C. DNA concentration was measured with the Qubit dsDNA HS Assay Kit (Q32854; Thermo Fisher Scientific).

### Library preparation and DNA sequencing

Twenty nanograms of DNA per sample was utilized as input for preparing fragment libraries with the NEBNext Ultra II DNA Library Prep Kit for Illumina (E7645L; New England Biolabs). All steps were performed according to the user manual E7645 (Version 5.0_06-2018; New England Biolabs), except that all reactions were scaled down to half the recommended volumes. DNA libraries were barcoded by unique dual indexes (8 bp) using the NEBNext Multiplex Oligos for Illumina (E6440S, E6442S, and E6448S; New England Biolabs). All generated DNA libraries were amplified by six PCR cycles, and libraries were quantified using the Qubit dsDNA HS Assay Kit (Q32854; Thermo Fisher Scientific). Library sizes were assessed using the Bioanalyzer High Sensitivity DNA Assay (5067-4626; Agilent Technologies). Equimolar amounts of individually barcoded libraries were pooled. The Illumina NovaSeq 6000 platform was used for sequencing (NovaSeq 6000 SP reagent kit v1.5, 100 cycles, single-end reads; Illumina; 20028401). Sequencing read quality control was performed with FastQC (79), and data integration and summary visualization were performed using MultiQC software (80).

### Taxonomic classification and normalization

Since nasal lavages were collected bilaterally at both baseline and V1, the proband’s samples from each timepoint were bioinformatically concatenated, enabling their treatment as a single representative sample per timepoint.

Next, a whole-metagenome sequencing alignment pipeline (41) was employed for taxonomic classification, using default settings optimized for single-end data. The reference database used is available for download at https://zenodo.org/records/20084561.

For each sample, raspir (version 1.0.2) (76) was used to filter out microbial species with nonuniform read distributions across the reference genome. The microbial reads were normalized to an ideal genome length of one million base pairs and a sequencing depth of one million reads, accounting for variations in both chromosome length and sequencing depth (reads per million base pair values). Subsequently, to estimate absolute microbial abundance, the relative abundance of each species was divided by the fraction of sequin-associated reads in each sample (40). Calculation of bacterial cells per human cell (41, 42) was used as an alternative approach for absolute abundance normalization.

### Statistical analysis

Statistical analyses of clinical parameters were conducted using SPSS software version 29.0.2.0. All other data analyses were performed in RStudio version 4.3.2 (2023-10-31) using R, including the vegan package for community ecology analysis (81) and the rcompanion package for statistical testing (82). The Wilcoxon signed-rank test (effect size *r*) was applied to compare the two dependent groups, baseline and V1. Confidence intervals (CI) were identified.

Microbial diversity within the CF nasal habitat was evaluated at both time points by analyzing relative and absolute abundances, Shannon and Simpson diversity indices, Pielou’s evenness index, and species richness. Beta diversity was assessed through principal coordinate analysis (PCoA) of Euclidean-distance matrices (83) derived from log10-scaled absolute microbial abundance counts. Multivariate dispersion between baseline and V1 samples was quantified by measuring the distance of individual samples from the group centroid in multivariate space. Relationships between metadata variables and the PCoA plot were examined using a permutation test (envfit, permutations = 999).

For the ecological network analysis, the best practice guidelines for species co-occurrence network construction were followed (44). Spearman’s rank correlation matrices were computed from absolute microbial abundance counts at each time point, and significant positive and negative correlations were extracted (Spearman’s *P* value ≤  0.01, correlation coefficient ≥ +0.2 or ≤ −0.2). The open-source software Gephi (84) was utilized for a directed network analysis with the continuous graph layout algorithm ForceAtlas (inertia = 0.1; repulsion strength = 5,000; attraction strength = 10.0; maximum displacement = 10.0; auto stabilize function = TRUE; autostab strength = 80.0; autostab sensibility = 0.2; gravity = 30.0; attraction distribution = TRUE; adjust by sizes = TRUE; speed = 1.0.) (85). The network parameters node authority, closeness centrality, and betweenness centrality were obtained.

Spearman’s rank-order correlations were assessed as a nonparametric ranked-based pairwise correlation test with a significance level of 0.05 (*). Significance tests with *P* values were calculated for each pair of input features, and *P* values were adjusted for multiple comparisons using the Benjamini-Hochberg correction.

## Data Availability

Microbial sequencing data are stored in the European Nucleotide Archive (PRJEB96177). Coding scripts and input files are available from https://github.com/irosenboom/Effects-of-ETI-on-the-nasal-metagenome-in-CF.

## AFTER EPUB

*[This article was published on 13 July 2026 with a typographical error in a grant number. The error was corrected in the current version, posted on 29 July 2026.]*

## SUPPLEMENTAL MATERIAL

Supplemental material spectrum.00601-26-s0001.docx Supplemental results, Tables S1 to S6, and Figures S1 to S3.

ASM does not own the copyrights to Supplemental Material that may be linked to, or accessed through, an article. The authors have granted ASM a non-exclusive, world-wide license to publish the Supplemental Material files. Please contact the corresponding author directly for reuse.
