# Supplementary material for: Effects of elexacaftor/tezacaftor/ivacaftor on the nasal microbial metagenome in cystic fibrosis
Source: Microbiol Spectr. 2026 Jul 13;14(8):e00601-26. doi: 10.1128/spectrum.00601-26 (PMC13435744; doi:10.1128/spectrum.00601-26)
Supplement: Supplementary file 1 [file spectrum.00601-26-s0001.docx]

**Online Supplementary Material**

**Effects of Elexacaftor/Tezacaftor/Ivacaftor on the Nasal Microbial Metagenome in Cystic Fibrosis**

**Tom L. Hertramph**^1,2^, Marie Dorda^3^, Sophia T. Pallenberg^1^, Annette Sauer-Heilborn^4^, Felix C. Ringshausen^4,5^, Matthias Steglich^3^, Gesine Hansen^1,5,6^, Burkhard Tümmler^1,5,6^, Lutz Wiehlmann^3^, Ilona Rosenboom^1,3,5^, Anna-Maria Dittrich^1,5,6#^

^1^Department for Pediatric Pneumology, Allergology and Neonatology, Hannover Medical School (MHH), Hannover, Germany

^2^DigiStrucMed Program, Dean’s Office for Academic Career Development, Hannover Medical School (MHH), Hannover, Germany
^3^Research Core Unit Genomics, Hannover Medical School (MHH), Hannover, Germany

^4^Department of Respiratory Medicine and Infectious Diseases, Hannover Medical School (MHH), Hannover, Germany
^5^German Center for Lung Research, Biomedical Research in Endstage and Obstructive Lung Disease Hannover (BREATH), Hannover Medical School (MHH), Hannover, Germany

^6^Cluster of Excellence RESIST (EXC 2155), German Research Foundation (DFG), Hannover Medical School (MHH), Hannover, Germany.

**Running Head:** Effects of ETI on Nasal Microbial Metagenome in CF

#Address correspondence to Anna-Maria Dittrich, Dittrich.Anna-Maria@mh-hannover.de.

**Supplementary Text**

**Evolution of the largest network component of species co-occurrence networks**

The evolution of the largest network component of species co-occurrence networks from baseline to 15 weeks (V1) of ETI revealed relatively stable taxonomic composition despite marked global network reorganization observed in the main analysis. Specifically, the dominant genera within the largest component (Figure 5A and B, red modularity class), primarily Neisseria spp.*,* Streptococcus spp., Rothia spp., and Gemella spp., remained largely consistent across both time points, with only minor shifts in relative abundance (Figure S1A). This indicates that ETI-driven changes in network architecture were not primarily driven by major compositional turnover within the core taxa.

Consistent with this observation, measures of within-component microbial diversity showed only modest variation between baseline and V1. Shannon diversity and Simpson diversity indices exhibited minimal changes (Figure S1B, C). Similarly, Pielou’s evenness index remained largely unchanged (Figure S1D). The number of genera within the largest component also showed only slight variation between time points (Figure S1E). Together, these findings support the notion that the internal ecological composition of the largest module is relatively resilient to ETI treatment.

The observed changes in statistical network metrics were closely aligned with the global reorganization of the network from a densely interconnected core at baseline to a less dense structure at V1 (Figure 5A–B). Kleinberg’s Authority scores remained largely unchanged from baseline to V1 (P = 0.722, median at baseline = 0.17, median at V1 = 0.16, Wilcoxon signed-rank test) (Figure S1F), indicating that incoming connections per node were relatively stable despite the overall reduction in network density. In contrast, betweenness centrality increased markedly at V1 (P < 0.0001, median at baseline = 0.0, median at V1 = 0.02, Wilcoxon signed-rank test) (Figure S1G), reflecting a greater reliance on key intermediary nodes as the dense core network transitioned to a more scattered structure. Conversely, closeness centrality decreased significantly (P < 0.0001, median at baseline = 0.93, median at V1 = 0.39, Wilcoxon signed-rank test) (Figure S1H), consistent with longer average path lengths in the less connected network at V1. Additional statistical metrics, including reductions in the number of interconnections, average degree, graph density, and the relative contribution of the largest network component from baseline to V1 (Table S3) further support this overall pattern.

**Individual Case Reports**

To further explore interindividual variability in microbial and clinical responses to ETI, heat tree analyses of absolute species abundances were performed in four representative CF patients with divergent trajectories (Figure S2, A – H). These analyses revealed marked heterogeneity in microbiome restructuring that paralleled differences in clinical outcomes.

In CF-02 (14 years, no CFTR modulator pre-treatment, no colonization with *P. aeruginosa*, colonization with *H. influenzae*), ETI treatment was associated with a pronounced expansion and diversification of the nasal microbiome (Shannon diversity, +2.59; Simpson diversity, +0.77; Pielou’s evenness, +0.62; Species number, +34) (Figure S2, A and B). This shift toward a more complex and evenly distributed community coincided with moderate improvements in lung function (FEV1, +9.71 %; MEF25, +45 %).

Conversely, CF-09 (14 years, no CFTR modulator pre-treatment, no colonization with *P. aeruginosa*, colonization with *H. influenzae*), with similar initial clinical situation, exhibited a reduction in microbial diversity (Shannon diversity, -1.11; Simpson diversity, -0.25; Pielou’s evenness, -0.14.; Species number, -25), accompanied by an increased absolute abundance of *H. influenzae* (+3.86). This shift was paralleled by a less favorable clinical trajectory under ETI (FEV1, +8.63 %; MEF25, -25 %) (Figure S2, C and D).

CF-14, representing a patient with advanced disease and chronic Pseudomonas aeruginosa colonization (44 years, no CFTR modulator pre-treatment, no colonization with *H. influenzae*), showed minimal changes in microbial composition following ETI (Shannon diversity, +0.17; Simpson diversity, +0.09; Pielou’s evenness, +0.13; Species number, +/-0) (Figure S2, E and F). The persistence of a simplified, pathogen-dominated microbiome was paralleled by only marginal clinical improvement (FEV1, +5.21 %; MEF25, +/- 0 %).

Finally, CF-24 (24 years, CFTR modulator pre-treatment with LUM/IVA, colonization with *P. aeruginosa*, colonization with *H. influenzae*) demonstrated a distinct pattern in which ETI led to the disappearance of key CF-associated pathogens *P. aeruginosa* and *H. influenzae*, alongside a shift toward skin-associated commensals *C. acnes* and *S. epidermidis* (Figure S2, G and H). Despite an overall decrease in diversity (Shannon diversity, -1.11; Simpson diversity, -0.33; Pielou’s evenness, -0.05; Species number, -9), this compositional shift was associated with substantial improvement in lung function (FEV1, +30.18 %; MEF25, +78 %).

Overall, these case-based analyses highlight that ETI-induced changes in the nasal microbiome are highly individualized and can manifest as diversification, contraction, or selective restructuring of microbial communities. Importantly, these patterns appear to reflect clinical response, supporting the link between microbiome dynamics and therapeutic outcome.

**BMI Percentiles**

To obtain a suitable parameter for body weight that was comparable across age groups, BMI percentiles were determined from body weight and height, using the calculator “BMI - Perzentile und SDS MVZ Labor Dortmund” (https://www.labor-dortmund.de/laborrechner/labor-rechner/bmi-perzentile-und-sds/). The calculator takes into account the age at the respective visit and the biological sex and the calculation is based on data from the German population-based cohort “Nationale Verzehrsstudie II” [1, 2], and the guidelines of the German “Arbeitsgemeinschaft Adipositas im Kindes- und Jugendalter (AGA)” (S3-Leitlinie „Therapie und Prävention der Adipositas im Kindes- und Jugendalter“; available at https://register.awmf.org/assets/guidelines/050-002l_S3_Therapie-Praevention-Adipositas-Kinder-Jugendliche_2019-11.pdf; AWMF-Nr. 050-002; Version August 2019).

**Supplementary Tables**

**TABLE S1** Patient characteristics and clinical data

| Patient | Gender | Age at Baseline (Years) | Genotype | Pre-Modulator | FEV_1_ (% predicted) | | MEF_25_ (% predicted) | | BMI  (Percentile) | | Sweat Chloride Concentration [mmol/l] | |  |
| --- | --- | --- | --- | --- | --- | --- | --- | --- | --- | --- | --- | --- | --- |
|  |  |  |  |  | Baseline | V1 | Baseline | V1 | Baseline | V1 | Baseline | V1 |  |
| CF-01 | f | 16,1 | F/MF | - | 83,4 | 94,4 | 80 | 103 | 65,5 | 75,8 | 106 | 20 |  |
| CF-02 | f | 14,7 | F/MF | - | 94,7 | 104,4 | 66 | 111 | 34,5 | 38,2 | 113 | 65 |  |
| CF-03 | m | 12,8 | F/MF | - | 86,1 | 114,0 | 53 | 128 | 6,7 | 21,2 | 115 | 50 |  |
| CF-04 | f | 21,3 | F/MF | - | 52,4 | 81,2 | 13 | 71 | 13,6 | 13,6 | 103 | 14 |  |
| CF-06 | f | 13,6 | F/F | - | 96,6 | 98,9 | 92 | 96 | 61,8 | 72,6 | 104 | 27 |  |
| CF-07 | m | 17,1 | F/MF | - | 75,5 | 103,0 | 36 | 106 | 38,2 | 38,2 | 108 | 90 |  |
| CF-08 | m | 13,3 | F/MF | - | 71,0 | 83,9 | 28 | 28 | 81,6 | 90,3 | 108 | 36 |  |
| CF-09 | f | 14,0 | F/MF | - | 74,3 | 82,7 | 99 | 74 | 18,4 | 34,5 | 104 | 25 |  |
| CF-10 | f | 13,8 | F/MF | - | 117,3 | 132,4 | 92 | 159 | 65,5 | 50,0 | 104 | 54 |  |
| CF-12 | f | 13,7 | F/MF | - | 100,1 | 114,5 | 73 | 106 | 57,9 | 84,1 | 110 | 40 |  |
| CF-13 | f | 12,7 | F/MF | - | 96,0 | 109,9 | 62 | 91 | 46,0 | 38,2 | 88 | 41 |  |
| CF-14 | m | 44,0 | F/MF | - | 77,4 | 82,7 | 22 | 22 | 65,5 | 75,8 | 112 | 53 |  |
| CF-15 | f | 20,8 | F/MF | - | 47,8 | 75,3 | 16 | 46 | 0,5 | 5,5 | 92 | 38 |  |
| CF-16 | m | 31,4 | F/F | - | 61,1 | 91,8 | 32 | 46 | 4,5 | 4,5 | 75 | 56 |  |
| CF-18 | m | 44,8 | F/F | TEZ/IVA | 57,2 | 59,2 | 19 | 22 | 5,5 | 11,5 | 108 | 52 |  |
| CF-19 | f | 12,1 | F/MF | - | 59,2 | 76,7 | 32 | 40 | 1,8 | 24,2 | 98 | 70 |  |
| CF-21 | m | 32,1 | F/F | - | 72,7 | 88,2 | 33 | 47 | 4,5 | 18,4 | 98 | 63 |  |
| CF-24 | f | 24,3 | F/F | LUM/IVA | 91,3 | 121,4 | 39 | 117 | 24,2 | 30,9 | 79 | 37 |  |
| CF-25 | m | 20,7 | F/MF | - | 78,4 | 95,2 | 49 | 67 | 15,9 | 30,9 | 86 | 53 |  |
| CF-26 | m | 20,4 | F/MF | - | 101,0 | 105,9 | 86 | 98 | 42,1 | 46,0 | 82 | 31 |  |
| CF-27 | f | 42,1 | F/F | LUM/IVA | 87,6 | 104,1 | 31 | 68 | 69,1 | 61,8 | 84 | 47 |  |
| CF-29 | m | 13,9 | F/F | - | 94,0 | 113,1 | 71 | 211 | 9,7 | 6,7 | 98 | 30 |  |
| CF-31 | f | 15,6 | F/MF | - | 101,2 | 112,3 | 87 | 116 | 18,4 | 18,4 | 99 | 44 |  |
| CF-33 | m | 19,2 | F/F | LUM/IVA | 97,2 | 96,7 | 95 | 91 | 61,8 | 61,8 | 104 | 64 |  |
| Abbreviations: f, female; m, male; F, Phe508del mutation; MF, minimal function mutation; LUM/IVA, lumacaftor/ivacaftor; TEZ/IVA, tezacaftor/ivacaftor; FEV1, forced expiratory volume in 1 second capacity in percent predicted; MEF25, mid expiratory flow at 25 % of vital capacity in percent predicted; BMI, body mass index. | | | | | | | | | | | | | |

**TABLE S2** Clinical, functional and microbial parameters of recruited, included and CFTR modulator treatment-naive CF patients at baseline and V1

| **Study parameters** | | | **Patients with existing clinical (n=27) and microbial data (n=26[baseline], n=27[V1])** | | | | | **Patients included in the study (n=24)** | | | | | **CFTR modulator treatment-naive patients included in the study (n=20)** | | | | |
| --- | --- | --- | --- | --- | --- | --- | --- | --- | --- | --- | --- | --- | --- | --- | --- | --- | --- |
|  |  |  | **Baseline** [median (range) or n (%)] | **V1** [median (range) or n (%)] | mean difference | 95 % CI | P value | **Baseline** [median (range) or n (%)] | **V1** [median (range) or n (%)] | mean difference | 95 % CI | P value | **Baseline** [median (range) or n (%)] | **V1** [median (range) or n (%)] | mean difference | 95 % CI | P value |
| No. of patients that gave informed consent (n) | | 29 |  |  |  |  |  |  |  |  |  |  |  |  |  |  |  |
| No. of patients with existing clinical data (n) | | 27 |  |  |  |  |  |  |  |  |  |  |  |  |  |  |  |
| No. of patients with existing microbial data (n) | | 26 (Baseline) |  |  |  |  |  |  |  |  |  |  |  |  |  |  |  |
|  |  | 27 (V1) |  |  |  |  |  |  |  |  |  |  |  |  |  |  |  |
| No. of patients included in analysis (n) | | 24 |  |  |  |  |  |  |  |  |  |  |  |  |  |  |  |
| No. of CFTR modulator treatment-naive patients (n) | | 20 |  |  |  |  |  |  |  |  |  |  |  |  |  |  |  |
|  |  |  |  |  |  |  |  |  |  |  |  |  |  |  |  |  |  |
| **Participant Characteristics and CFTR Modulator Exposure** | | |  | |  |  |  |  |  |  |  |  |  |  |  |  |  |
|  | Age (years) |  | 17.13 (12.10 to 44.81) |  |  |  |  | 16.60 (12.10 to 44.81) |  |  |  |  | 15.15 (12.10 to 44.00) |  |  |  |  |
|  | Sex (female) |  | 15 (55.6 %) |  |  |  |  | 13 (54 %) |  |  |  |  | 11 (55 %) |  |  |  |  |
|  | Phe508del homozygous |  | 10 (37.0 %) |  |  |  |  | 8 (33 %) |  |  |  |  | 4 (20 %) |  |  |  |  |
|  | Phe508del heterozygous |  | 17 (63.0 %) |  |  |  |  | 16 (67 %) |  |  |  |  | 16 (80 %) |  |  |  |  |
|  | CFTR-modulator pre-treatment |  | 5 (18.5 %) |  |  |  |  | 4 (17 %) |  |  |  |  | 0 (0 %) |  |  |  |  |
|  | LUM/IVA |  | 3 (11.1 %) |  |  |  |  | 3 (13 %) |  |  |  |  | 0 (0 %) |  |  |  |  |
|  | TEZ/IVA |  | 2 (7.4 %) |  |  |  |  | 1 (4 %) |  |  |  |  | 0 (0 %) |  |  |  |  |
|  | Time period baseline to V1 (days) |  | 109 (46 to 148) |  |  |  |  | 109 (46 to 148) |  |  |  |  | 109.5 (46 to 148) |  |  |  |  |
|  |  |  |  |  |  |  |  |  |  |  |  |  |  |  |  |  |  |
| **Clinical parameters** | |  |  | |  |  |  |  |  |  |  |  |  |  |  |  |  |
|  | FEV1 (%) |  | 83.40 (47.80 to 117.32) | 96.73 (59.19 to 132.43) |  |  |  | 84.76 (47.80 to 117.32) | 97.82 (59.19 to 132.43) |  |  |  | 80.89 (47.81 to 117.32) | 97.06 (75.34 to 132.43) |  |  |  |
|  | ∆ FEV1 (%) to baseline |  |  | +14.32 (-0.48 to 33.73) | 15.50 | 11.61; 19.39 | < 0.001 |  | +14.71 (-0.48 to 30.72) | 15.35 | 11.27; 19.34 | < 0.001 |  | +14.71 (2.35 to 30.72) | 15.35 | 12.01; 20.02 | < 0.001 |
|  |  |  |  |  |  |  |  |  |  |  |  |  |  |  |  |  |  |
|  | MEF25 (%) |  | 49 (13 to 99) | 91 (22 to 211) |  |  |  | 51 (13 to 99) | 91 (22 to 211) |  |  |  | 57.5 (13 to 99) | 93.5 (22 to 211) |  |  |  |
|  | ∆ MEF25 (%) to baseline |  |  | +29 (-25 to 140) | 32.85 | 19.00; 46.71 | < 0.001 |  | +26 (-25 to 140) | 31.58 | 16.51; 46.66 | < 0.001 |  | +26 (-25 to 140) | 32.2 | 15.21; 49.19 | < 0.001 |
|  |  |  |  |  |  |  |  |  |  |  |  |  |  |  |  |  |  |
|  | LCI (CEV/FRC) |  | 11.65 (7.62 to 21.91) | 8.74 (6.86 to 18.72) |  |  |  | 11.245 (8.06 to 21.57) | 8.465 (7.26 to 18.72) |  |  |  | 10.62 (8.06 to 21.57) | 8.11 (6.86 to 18.72) |  |  |  |
|  | ∆ LCI to baseline |  |  | -1.99 (-5.34 to 3.58) | -1.73 | -2.93; -0.52 | 0.008 |  | -2.215 (-5.34 to 1.25) | -2.061 | -3.11; -1.01 | < 0.001 |  | -2.22 (-4.55 to 1.25) | -2.01 | -3.03; -0.99 | < 0.001 |
|  |  |  |  |  |  |  |  |  |  |  |  |  |  |  |  |  |  |
|  | Sweat chloride (mmol/L) |  | 103 (75 to 115) | 47 (10 to 101) |  |  |  | 103.5 (75 to 115) | 45.5 (14 to 90) |  |  |  | 103.5 (75 to 115) | 42.5 (14 to 90) |  |  |  |
|  | ∆ Sweat chloride (mmol/L) to baseline |  |  | -51 (-89 to -8) | -52.19 | -60.79; -43.58 | < 0.001 |  | -52.5 (-89 to -18) | -53.25 | -61.63; -44.87 | < 0.001 |  | -54.5 (-89 to -18) | -55.15 | -64.99; -45.30 | < 0.001 |
|  |  |  |  |  |  |  |  |  |  |  |  |  |  |  |  |  |  |
|  | Leucocytes (thous./ µl) |  | 7.6 (5.0 to 10.5) | 6.2 (4.8 to 11.9) |  |  |  | 7.8 (5.0 to 10.5) | 6.2 (4.8 to 11.9) |  |  |  | 7.8 (5.4 to 10.5) | 6.7 (5.0 to 11.9) |  |  |  |
|  | ∆ Leucocytes (thous./ µl) to baseline |  |  | -1.1 (-3.4 to 3.1) | -0.61 | -1.37; 0.15 | 0.108 |  | -0.95 (-3.4 to 3.1) | -0.64 | -1.50; 0.21 | 0.132 |  | -1.1 (-3.4 to 3.1) | -0.64 | -1.50; 0.21 | 0.132 |
|  |  |  |  |  |  |  |  |  |  |  |  |  |  |  |  |  |  |
|  | BMI percentile |  | 24.2 (0.5 to 88.5) | 34.5 (4.5 to 91.9) |  |  |  | 29.4 (0.5 to 81.1) | 36.4 (4.5 to 90.3) |  |  |  | 26.45 (0.5 to 81.6) | 36.35 (4.5 to 90.3) |  |  |  |
|  | ∆ BMI percentile to baseline |  |  | +6.0 (-15.5 to 26.2) | 6.27 | 2.51; 10.03 | 0.002 |  | +5.5 (-15.5 to 26.2) | 5.83 | 1.73; 9.92 | 0.007 |  | +6.85 (-15.5 to 26.2) | 6.73 | 1.99; 11.45 | 0.008 |
|  |  |  |  |  |  |  |  |  |  |  |  |  |  |  |  |  |  |
| **Metagenome diversity parameters (all samples)** | |  |  | |  |  |  |  |  |  |  |  |  |  |  |  |  |
|  | Species richness (n) |  | 4 (1 to 42) | 7 (2 to 44) |  |  |  | 4.5 (2 to 42) | 7 (2 to 44) |  |  |  | 4.5 (2 to 42) | 8 (2 to 44) |  |  |  |
|  | Pielou's evenness index |  | 0.76 (0.08 to 1.00)* | 0.65 (0.18 to 0.94) |  |  |  | 0.76 (0.08 to 1.00) | 0.65 (0.18 to 0.94) |  |  |  | 0.77 (0.08 to 1.0) | 0.64 (0.18 to 0.94) |  |  |  |
|  | Shannon diversity index |  | 0.92 (0.00 to 2.95) | 1.15 (0.20 to 3.22) |  |  |  | 1.02 (0.09 to 2.95) | 1.19 (0.20 to 2.98) |  |  |  | 0.94 (0.09 to 2.95) | 1.28 (0.20 to 2.98) |  |  |  |
|  | Simpson diversity index |  | 0.52 (0.00 to 0.92) | 0.59 (0.08 to 0.94) |  |  |  | 0.57 (0.03 to 0.92) | 0.61 (0.08 to 0.92) |  |  |  | 0.55 (0.03 to 0.92) | 0.66 (0.08 to 0.92) |  |  |  |
|  |  |  |  |  |  |  |  |  |  |  |  |  |  |  |  |  |  |
| **Bacterial DNA load** | |  |  | |  |  |  |  |  |  |  |  |  |  |  |  |  |
|  | Total |  | 0.62 (0.03 to 10.10) | 1.70 (0.05 to 11.07) |  |  |  | 0.64 (0.04 to 10.1) | 1.66 (0.05 to 11.07) |  |  |  | 0.67 (0.04 to 10.1) | 1.66 (0.08 to 11.07) |  |  |  |
|  | *P.aeruginosa* (in baseline/V1 positive patients) |  | 0.04 (0.003 to 0.67) | 0 (0 to 0.55) |  |  |  | 0.04 (0.003 to 0.676) | 0.00 (0.00 to 0.17) |  |  |  | 0.05 (0.002 to 0.67) | 0 (0 to 0.17) |  |  |  |
|  | *H. influenzae* (in baseline/V1 positive patients) |  | 0.10 (0.00 to 1.09) | 0.00 (0.00 to 4.95) |  |  |  | 0.10 (0.00 to 1.09) | 0.00 (0.00 to 4.95) |  |  |  | 0.1 (0 to 1.09) | 0.02 (0 to 4.95) |  |  |  |
|  | *S. aureus* (in baseline/V1 positive patients) |  | 0.00 (0.00 to 0.14) | 0.04 (0.00 to 2.98) |  |  |  | 0.00 (0.00 to 0.14) | 0.05 (0.00 to 2.98) |  |  |  | 0 (0 to 0.12) | 0.04 (0 to 2.98) |  |  |  |
| Abbreviations: LUM/IVA, luacaftor/ivacaftor; TEZ/IVA, tezacaftor/ivacaftor; FEV1, forced expiratory volume in 1 second capacity in percent predicted; MEF25, mid expirtory flow at 25 % of vital capacity in percent predicted; LCI, lung clearance index; BMI, body mass index. Note: The bacterial DNA load is based on sequin-normalized data, calculated as the rario of RPMM values (number of reads per million base pairs of reference genome and million sequencing reads) assigned to each specis to the RPMM values of the spiked-in internal reference standard (sequins). *For analyses of the dataset comprising all available microbial data, baseline Pielou’s evenness values were calculated from 25 patient samples, as one sample contained only a single species, rendering the metric undefined. | | | | | | | | | | | | | | | | | |

**TABLE S3** Statistical metrics of baseline and V1 cooccurrence networks

| **Network parameters** | **Baseline** | **V1** |
| --- | --- | --- |
| Number of nodes (in total) | 68 | 81 |
| Number of edges (in total) | 775 | 486 |
| Average degree | 22.8 | 12.0 |
| Graph density | 0.34 | 0.15 |
| Connected components | 4 | 1 |
| Average path length | 1.3 | 3.2 |
| Number of modularity classes | 5 | 6 |
| Average clustering coefficient | 0.85 | 0.55 |
| Contribution of the largest module | 60.29% | 29.63% |
| Average degree (largest module) | 35.3 | 15.9 |
| Note: input arguments for running the Force Atlas algorithm were as follows: inertia = 0.1; repulsion strength = 5,000; attraction strength = 10.0; maximum displacement = 10.0; auto stabilize function = TRUE; autostab strength = 80.0; autostab sensibility = 0.2; gravity = 30.0; attraction distribution = TRUE; adjust by sizes = TRUE; speed = 1.0.  Network parameters are explained in the following. Number of nodes: The total count of species in the network. Number of edges: The total count of links between the nodes. Average degree: The average number of connections per node. Graph density: The ratio of existing edges to the maximum possible edges. Connected components: The number of independent, disconnected subgraphs in the network. Average path length: The average number of steps required to go from any node to any other node. Number of modularity classes: The total number of distinct clusters. Average clustering coefficient: The likelihood that two nodes connected to a third node are also connected to each other. Contribution of the largest module: The percentage of all nodes that belong to the largest identified community. Average degree (largest module): The average number of connections for only the nodes within the largest community. | | |

**TABLE S4** Species composition of modularity classes in baseline and V1 co-occurrence networks

| Baseline | | | First study visit (V1) | | |
| --- | --- | --- | --- | --- | --- |
| Modularity class | Genus | Species | Modularity class | Genus | Species |
| red | *Abiotrophia* | *Abiotrophia defectiva* | red | *Actinomyces* | *Actinomyces oris* |
|  | *Actinomyces* | *Actinomyces naeslundii* |  | *Aggregatibacter* | *Aggregatibacter aphrophilus* |
|  |  | *Actinomyces oris* |  | *Capnocytophaga* | *Capnocytophaga sputigena* |
|  | *Aggregatibacter* | *Aggregatibacter aphrophilus* |  | *Enterococcus* | *Enterococcus faecalis* |
|  | *Capnocytophaga* | *Capnocytophaga gingivalis* |  | *Fusobacterium* | *Fusobacterium periodonticum* |
|  |  | *Capnocytophaga leadbetteri* |  | *Gemella* | *Gemella haemolysans* |
|  |  | *Capnocytophaga ochracea* |  |  | *Gemella morbillorum* |
|  |  | *Capnocytophaga sputigena* |  |  | *Gemella sanguinis* |
|  | *Fusobacterium* | *Fusobacterium hwasookii* |  | *Lautropia* | *Lautropia mirabilis* |
|  |  | *Fusobacterium nucleatum* |  | *Neisseria* | *Neisseria mucosa* |
|  | *Gemella* | *Gemella haemolysans* |  |  | *Neisseria subflava* |
|  |  | *Gemella morbillorum* |  | *Prevotella* | *Prevotella denticola* |
|  |  | *Gemella sanguinis* |  |  | *Prevotella enoeca* |
|  | *Haemophilus* | *Haemophilus parahaemolyticus* |  | *Rhodococcus* | *Rhodococcus erythropolis* |
|  |  | *Haemophilus parainfluenzae* |  | *Rothia* | *Rothia aeria* |
|  | *Lautropia* | *Lautropia mirabilis* |  | *Staphylococcus* | *Staphylococcus argenteus* |
|  | *Neisseria* | *Neisseria cinerea* |  |  | *Staphylococcus warneri* |
|  |  | *Neisseria elongata* |  | *Stenotrophomonas* | *Stenotrophomonas maltophilia* |
|  |  | *Neisseria mucosa* |  | *Streptococcus* | *Streptococcus cristatus* |
|  |  | *Neisseria subflava* |  |  | *Streptococcus gordonii* |
|  | *Prevotella* | *Prevotella denticola* |  |  | *Streptococcus mitis* |
|  |  | *Prevotella enoeca* |  |  | *Streptococcus oralis* |
|  |  | *Prevotella melaninogenica* |  |  | *Streptococcus pneumoniae* |
|  | *Pseudopropionibacterium* | *Pseudopropionibacterium propionicum* |  |  | *Streptococcus pseudopneumoniae* |
|  | *Rothia* | *Rothia aeria* | green | *Capnocytophaga* | *Capnocytophaga gingivalis* |
|  |  | *Rothia dentocariosa* |  | *Mycobacterium* | *Mycobacterium abscessus* |
|  |  | *Rothia mucilaginosa* |  | *Prevotella* | *Prevotella jejuni* |
|  | *Schaalia* | *Schaalia odontolytica* |  |  | *Prevotella melaninogenica* |
|  | *Streptococcus* | *Streptococcus constellatus* |  | *Pseudomonas* | *Pseudomonas denitrificans* |
|  |  | *Streptococcus equinus* |  | *Pseudopropionibacterium* | *Pseudopropionibacterium propionicum* |
|  |  | *Streptococcus gordonii* |  | *Rothia* | *Rothia dentocariosa* |
|  |  | *Streptococcus intermedius* |  |  | *Rothia mucilaginosa* |
|  |  | *Streptococcus mitis* |  | *Schaalia* | *Schaalia odontolytica* |
|  |  | *Streptococcus oralis* |  | *Streptococcus* | *Streptococcus equinus* |
|  |  | *Streptococcus parasanguinis* |  |  | *Streptococcus parasanguinis* |
|  |  | *Streptococcus pneumoniae* |  |  | *Streptococcus salivarius* |
|  |  | *Streptococcus pseudopneumoniae* |  |  | *Streptococcus sanguinis* |
|  |  | *Streptococcus sanguinis* |  | *Veillonella* | *Veillonella atypica* |
|  | *Veillonella* | *Veillonella atypica* |  |  | *Veillonella dispar* |
|  |  | *Veillonella dispar* |  |  | *Veillonella parvula* |
|  |  | *Veillonella parvula* | purple | *Campylobacter* | *Campylobacter ureolyticus* |
| green | *Achromobacter* | *Achromobacter denitrificans* |  | *Citrobacter* | *Citrobacter koseri* |
|  | *Campylobacter* | *Campylobacter ureolyticus* |  | *Corynebacterium* | *Corynebacterium aurimucosum* |
|  | *Citrobacter* | *Citrobacter koseri* |  |  | *Corynebacterium camporealensis* |
|  | *Cutibacterium* | *Cutibacterium acnes* |  |  | *Corynebacterium simulans* |
|  |  | *Cutibacterium avidum* |  |  | *Corynebacterium singulare* |
|  | *Escherichia* | *Escherichia coli* |  |  | *Corynebacterium striatum* |
|  |  | *Escherichia fergusonii* |  | *Cutibacterium* | *Cutibacterium avidum* |
|  | *Finegoldia* | *Finegoldia magna* |  | *Escherichia* | *Escherichia coli* |
|  | *Klebsiella* | *Klebsiella aerogenes* |  |  | *Escherichia fergusonii* |
|  | *Lawsonella* | *Lawsonella clevelandensis* |  | *Finegoldia* | *Finegoldia magna* |
|  | *Parvimonas* | *Parvimonas micra* |  | *Klebsiella* | *Klebsiella aerogenes* |
|  | *Proteus* | *Proteus vulgaris* |  | *Lawsonella* | *Lawsonella clevelandensis* |
|  | *Staphylococcus* | *Staphylococcus capitis* |  | *Proteus* | *Proteus vulgaris* |
|  |  | *Staphylococcus haemolyticus* |  | *Staphylococcus* | *Staphylococcus haemolyticus* |
| purple | *Corynebacterium* | *Corynebacterium aurimucosum* | pink | *Abiotrophia* | *Abiotrophia defectiva* |
|  |  | *Corynebacterium camporealensis* |  | *Campylobacter* | *Campylobacter curvus* |
|  |  | *Corynebacterium simulans* |  | *Capnocytophaga* | *Capnocytophaga leadbetteri* |
|  |  | *Corynebacterium singulare* |  | *Fusobacterium* | *Fusobacterium hwasookii* |
|  |  | *Corynebacterium striatum* |  |  | *Fusobacterium nucleatum* |
|  | *Enterococcus* | *Enterococcus faecalis* |  | *Haemophilus* | *Haemophilus influenzae* |
|  | *Rhodococcus* | *Rhodococcus erythropolis* |  |  | *Haemophilus parahaemolyticus* |
| pink | *Acinetobacter* | *Acinetobacter johnsonii* |  |  | *Haemophilus parainfluenzae* |
|  | *Micrococcus* | *Micrococcus luteus* |  | *Moraxella* | *Moraxella osloensis* |
|  | *Moraxella* | *Moraxella osloensis* |  | *Neisseria* | *Neisseria elongata* |
|  | *Staphylococcus* | *Staphylococcus hominis* |  | *Paracoccus* | *Paracoccus yeei* |
| yellow | *Bacteroides* | *Bacteroides fragilis* | yellow | *Actinomyces* | *Actinomyces naeslundii* |
|  |  | *Bacteroides vulgatus* |  | *Bifidobacterium* | *Bifidobacterium longum* |
|  |  |  |  | *Campylobacter* | *Campylobacter concisus* |
|  |  |  |  | *Lactobacillus* | *Lactobacillus amylovorus* |
|  |  |  |  | *Lactococcus* | *Lactococcus lactis* |
|  |  |  |  | *Neisseria* | *Neisseria cinerea* |
|  |  |  |  | *Serratia* | *Serratia marcescens* |
|  |  |  |  | *Xanthomonas* | *Xanthomonas campestris* |
|  |  |  | rose | *Cutibacterium* | *Cutibacterium acnes* |
|  |  |  |  | *Micrococcus* | *Micrococcus luteus* |
|  |  |  |  | *Staphylococcus* | *Staphylococcus epidermidis* |
|  |  |  |  |  | *Staphylococcus hominis* |
|  |  |  |  | *Streptococcus* | *Streptococcus anginosus* |
|  |  |  |  |  | *Streptococcus constellatus* |
|  |  |  |  |  | *Streptococcus intermedius* |

**TABLE S5** List of low abundant species, summarized as ‘others’, contributing to 10% of total abundance of all species in all samples ordered by decreasing total abundance*

| **Species name** | | |
| --- | --- | --- |
| *Haemophilus parainfluenzae* | *Neisseria cinerea* | *Capnocytophaga leadbetteri* |
| *Neisseria mucosa* | *Fusobacterium periodonticum* | *Actinomyces oris* |
| *Streptococcus parasanguinis* | *Prevotella enoeca* | *Lactococcus lactis* |
| *Parvimonas micra* | *Corynebacterium singulare* | *Bifidobacterium longum* |
| *Veillonella dispar* | *Stenotrophomonas maltophilia* | *Lawsonella clevelandensis* |
| *Lautropia mirabilis* | *Capnocytophaga gingivalis* | *Gemella morbillorum* |
| *Rothia mucilaginosa* | *Proteus vulgaris* | *Fusobacterium hwasookii* |
| *Neisseria subflava* | *Micrococcus luteus* | *Campylobacter curvus* |
| *Staphylococcus hominis* | *Streptococcus salivarius* | *Lactobacillus amylovorus* |
| *Cutibacterium avidum* | *Streptococcus anginosus* | *Actinomyces naeslundii* |
| *Streptococcus sanguinis* | *Schaalia odontolytica* | *Corynebacterium simulans* |
| *Streptococcus pseudopneumoniae* | *Streptococcus equinus* | *Xanthomonas campestris* |
| *Gemella haemolysans* | *Capnocytophaga sputigena* | *Corynebacterium aurimucosum* |
| *Achromobacter denitrificans* | *Pseudopropionibacterium propionicum* | *Escherichia fergusonii* |
| *Moraxella osloensis* | *Rhodococcus erythropolis* | *Corynebacterium camporealensis* |
| *Abiotrophia defectiva* | *Finegoldia magna* | *Staphylococcus xylosus* |
| *Enterococcus faecalis* | *Campylobacter concisus* | *Mycobacterium abscessus* |
| *Streptococcus intermedius* | *Haemophilus parahaemolyticus* | *Paracoccus yeei* |
| *Staphylococcus argenteus* | *Corynebacterium striatum* | *Capnocytophaga ochracea* |
| *Gemella sanguinis* | *Streptococcus gordonii* | *Streptococcus cristatus* |
| *Aggregatibacter aphrophilus* | *Prevotella jejuni* | *Prevotella denticola* |
| *Streptococcus pneumoniae* | *Bacteroides vulgatus* | *Serratia marcescens* |
| *Acinetobacter johnsonii* | *Fusobacterium nucleatum* | *Staphylococcus warneri* |
| *Neisseria elongata* | *Bacteroides fragilis* | *Pseudomonas denitrificans* |
| *sorted in descending order first by columns and then rows | | |

**TABLE S6** Programs for cell lysis (program 1) and DNA fragmentation (program 2)

using the Covaris S220 Focused-ultrasonicator

|  | **Program 1** | **Program 2** |
| --- | --- | --- |
|  | **Cell lysis** | **DNA Fragmentation** |
| Required run time | 3min 30 sec | 55 sec |
| Temperature | 5.0 – 8.0°C | 5.0 – 25.0°C |
|  | Begin repeat (6x) | Begin |
|  | 1. Treatment (5 sec) | 1. Treatment |
| Peak power | 200 | 140 |
| duty factor | 2.0 | 10.0 |
| cycles/burst | 100 | 200 |
|  | 2. Treatment (30 sec) | End |
| Peak power | 275.0 |  |
| duty factor | 5.0 |  |
| cycles/burst | 100 |  |
|  | End repeat |  |

**Supplementary Figures and Figure Legends**

**FIGURE S1** Evolution the largest network component of species co-occurrence networks (Figure 5A and B, red modularity class) from CF nasal lavage samples at baseline and after 15 weeks (V1) of ETI. (**A**) Stacked bar-plot depicting the mean species abundance (in %). (**B**, **C**, **D**, **E**) Presentation of changes in the microbial diversity, based on the Shannon diversity index (B), the Simpson diversity index (C), the Pielou's evenness index (D), and the number of genera (E). (**F**) Kleinberg’s Authority (Wilcoxon adjusted P value [P] = 0.722, r = 0.045, CI = -0.0455 – 0.0151). The Authority score of a node in a network indicates the total number of incoming edges to that node. (**G**) Betweenness centrality (P < 0.0001, r = 0.621, CI = 0.0138 – 0.0284). The betweenness centrality of a node quantifies the number of shortest paths in the graph that traverse through that node. (**H**) Closeness centrality (P < 0.0001, r = 0.852, CI = -0.5333 – -0.4762). The Closeness centrality score of a node reflects its average shortest distance to all other vertices in the network. Note: input arguments for running the Force Atlas algorithm were as follows: inertia = 0.1; repulsion strength = 5,000; attraction strength = 10.0; maximum displacement = 10.0; auto stabilize function = TRUE; autostab strength = 80.0; autostab sensibility = 0.2; gravity = 30.0; attraction distribution = TRUE; adjust by sizes = TRUE; speed = 1.0. During data preprocessing, the significance level for extracting correlations was set at P < 0.01. ns, not significant; ****, P < 0.0001.

**FIGURE S2** Heat tree representation of absolute species abundances in four diverging CF patients (1^st^ row CF-02, A & B; 2^nd^ row CF-09, C & D; 3^rd^ row CF-14, E & F; 4^th^ row CF-24, G & H) baseline (A, C, E, G) and after 15-weeks of treatment with ETI (V1) (B, D, F, H). Note: Absolute abundance values are based on sequin-normalized data, calculated as the ratio of RPMM values (number of reads per million base pairs of reference genome and million sequencing reads) assigned to each species to the RPMM values of the spiked-in internal reference standard (sequins). Heat trees were generated by the Wochenende Pipeline. Absolute abundance in a single sample is indicated by color, whereas absolute abundance across all samples is represented by the size of the species name.

**FIGURE S3** Number of reads per sample in patient and negative control samples after processing with Wochenende. Nasal lavage samples yielded a median of 15.4 million sequencing reads, while negative blank controls yielded a median of 35.5 reads.

**Supplementary References**

1. C. Hemmelmann SB, M. Vens, J. Hebebrand, A. Ziegler. Perzentilen des Body-Mass-Index auch für 18- bis 80-Jährige? Daten der Nationalen Verzehrsstudie II. Dtsch Med Wochenschr 2010: 135: 848–852.

2. K. Kromeyer-Hauschild MW, D.Kunze, F. Geller, H. C.Geiß, V.Hesse, A. von Hippel, U. Jaeger, D.Johnsen, W.Korte, K.Menner, G. Müller, J.M.Müller, A. Niemann-Pilatus, T.Remer, F. Schaefer, H.-U.Wittchen, S. Zabransky, K.Zellner, A. Ziegler, J.Hebebrand. Perzentile für den Body-mass-Index für das Kindes- und Jugendalter unter Heranziehung verschiedener deutscher Stichproben. Monatsschr Kinderheilkd 2001: 149: 807–818.
